# RhoA‐ROCK competes with YAP to regulate amoeboid breast cancer cell migration in response to lymphatic‐like flow

**DOI:** 10.1096/fba.2021-00055

**Published:** 2022-02-14

**Authors:** Amina Mohammadalipour, Miguel F. Diaz, Megan Livingston, Adesuwa Ewere, Allen Zhou, Paulina D. Horton, Loretta T. Olamigoke, John M. Lamar, John P. Hagan, Hyun J. Lee, Pamela L. Wenzel

**Affiliations:** ^1^ Department of Integrative Biology & Pharmacology The University of Texas Health Science Center at Houston Texas USA; ^2^ Children’s Regenerative Medicine Program Department of Pediatric Surgery The University of Texas Health Science Center at Houston Texas USA; ^3^ Center for Stem Cell and Regenerative Medicine Brown Foundation Institute of Molecular Medicine The University of Texas Health Science Center at Houston Texas USA; ^4^ Biochemistry and Cell Biology Program MD Anderson UTHealth Graduate School of Biomedical Sciences The University of Texas Houston Texas USA; ^5^ School of Medicine University of Texas Medical Branch Galveston Texas USA; ^6^ Immunology Program MD Anderson UTHealth Graduate School of Biomedical Sciences The University of Texas Houston Texas USA; ^7^ Vivian L. Smith Department of Neurosurgery The University of Texas Health Science Center at Houston Texas USA; ^8^ Molecular and Cellular Physiology Albany Medical College Albany New York USA; ^9^ Department of Anatomy and Cell Biology College of Medicine Chung‐Ang University Seoul South Korea; ^10^ Department of Global Innovative Drugs Graduate School of Chung‐Ang University Seoul South Korea

**Keywords:** actomyosin cytoskeleton, biomechanical force, ECM–receptor interactions, hippo, mechanotransduction, motility, shear stress

## Abstract

Lymphatic drainage generates force that induces prostate cancer cell motility via activation of Yes‐associated protein (YAP), but whether this response to fluid force is conserved across cancer types is unclear. Here, we show that shear stress corresponding to fluid flow in the initial lymphatics modifies taxis in breast cancer, whereas some cell lines use rapid amoeboid migration behavior in response to fluid flow, a separate subset decrease movement. Positive responders displayed transcriptional profiles characteristic of an amoeboid cell state, which is typical of cells advancing at the edges of neoplastic tumors. Regulation of the HIPPO tumor suppressor pathway and YAP activity also differed between breast subsets and prostate cancer. Although subcellular localization of YAP to the nucleus positively correlated with overall velocity of locomotion, YAP gain‐ and loss‐of‐function demonstrates that YAP inhibits breast cancer motility but is outcompeted by other pro‐taxis mediators in the context of flow. Specifically, we show that RhoA dictates response to flow. GTPase activity of RhoA, but not Rac1 or Cdc42 Rho family GTPases, is elevated in cells that positively respond to flow and is unchanged in cells that decelerate under flow. Disruption of RhoA or the RhoA effector, Rho‐associated kinase (ROCK), blocked shear stress–induced motility. Collectively, these findings identify biomechanical force as a regulator amoeboid cell migration and demonstrate stratification of breast cancer subsets by flow‐sensing mechanotransduction pathways.

**Abbreviations**
ECM: extracellular matrix
LIMK: lim domain kinase
ROCK: rho‐associated kinase
WSS: wall shear stress
YAP: yes‐associated protein

## 1 INTRODUCTION

Metastasis, or tumor cell dissemination, is a major determinant of prognosis and risk of mortality. When cancer cells spread to and grow at secondary sites in the affected individual, they can impinge on organ function and contribute to cachexia, a devastating muscle‐wasting disease.^1^ During malignant transformation, the microenvironment surrounding a solid tumor can contribute to cancer cell survival, growth, and metastasis.^2^, ^3^ Changes in cellular composition, tissue density, interstitial flow, intratumoral pressure, and vasculature can all affect the biochemical and biomechanical properties of the tumor and surrounding tissue. Emerging evidence shows that the biophysical features of the microenvironment, such as stiffness of cells and extracellular matrix (ECM), nanotopography, and extracellular force,^4^, ^5^, ^6^, ^7^ can affect carcinogenesis and the spread of cancer.^8^, ^9^, ^10^ Progressive change in the architecture and mechanics of the ECM can promote cancer progression, metastasis, and chemoresistance.^11^ Indeed, stiffening of the ECM, and the resulting increased cellular contractility, promotes the ability of invading cells to penetrate the basement membrane and increases the risk for metastasis, recurrence, and poor prognosis.^9^, ^10^, ^12^ Thus, cellular mechanics appear to play an important role not only in normal physiology but also tumor progression and metastasis.

Carcinomas like breast cancer preferentially spread initially from the primary tumor via the lymphatic system, the vascular network that drains interstitial fluid into regional lymph nodes.^5^ Because lymph node metastasis is a major prognostic factor in breast cancer patient morbidity and mortality, standard of care for these patients includes lymphadenectomy of the sentinel lymph nodes for disease staging and disruption of metastatic dissemination.^13^ Despite the clinical significance of lymph node metastases, the mechanisms leading to dissemination of malignant cells through the lymphatics are poorly understood.^14^ Fluid flow in and around solid tumors influences extracellular gradients of growth factors and chemokines, cytokine production, immune cell adhesion, tumor antigen transport, and delivery of chemotherapeutic agents.^15^, ^16^ Flow also creates a frictional force, or wall shear stress (WSS), within the interstitial space of draining tumors and in the lymph nodes and lymphatic vessels.^17^, ^18^ Thus, it is critical to understand how biomechanical cues in the tumor microenvironment and lymphatic egress routes contribute to cancer progression and treatment resistance.^11^, ^19^

Shear stress generated by fluid drainage in initial lymphatics is estimated to be ≤0.2–1 dyne cm^−2^, whereas force in lymphatic collector ducts is pulsatile and can be as high as 5 dyne cm^−2^ at the vessel wall, within range of venous flow intensities.^20^, ^21^ In our prior study of prostate cancer, we demonstrated that WSS generated by fluid flow around tumor cells facilitates metastatic behavior.^18^ To evaluate the impact of fluid force on cancer cells, we used a microfluidics platform capable of exposing cultured cells to precisely controlled, low‐intensity laminar fluid shear stress recapitulating fluid movement over tumor cells entering the initial lymphatics. Using this platform, lymphatic‐like fluid flow can be modeled to mimic a major cancer cell egress route. WSS characteristic of flow within the lymphatic vasculature modified prostate cancer cell motility via regulation of a transcriptional cofactor, Yes‐associated protein (YAP), that has proto‐oncogenic properties and is a negative effector of the HIPPO tumor suppressor pathway. Activation of YAP has previously been shown to promote tumor metastasis via localization to the nucleus and interaction with the TEAD transcription factors.^22^ We showed that WSS induced activating dephosphorylation of YAP that permitted its localization to the nucleus, resulting in the upregulation of a network of genes with roles in cell migration, proliferation, and survival.^18^ YAP activation was required for enhanced cellular motility under WSS, through the Rho‐associated kinase (ROCK)‐Lim domain kinase (LIMK)‐Cofilin axis. Fluid force induced extensive development of filopodia and lamellipodia structures in the highly metastatic PC3 human prostate cancer cell line, further supporting that biophysical cues can enhance metastatic features of prostate cancer. Flow also increased secretion of metalloproteases, including MMP‐2 and MMP‐9, two gelatinases that degrade the most abundant component of the basement membrane, type IV collagen. Notably, MMP‐2 activity is linked to poor prognosis in breast, ovarian, prostate, lung, melanoma, and colorectal cancers,^23^ and MMP‐9 actively contributes to tumor progression and metastasis of triple‐negative breast cancer.^24^

We initiated the current study to define the role of lymphatic force in the regulation of metastatic behavior of breast cancer cells. Specifically, we evaluated whether flow‐induced signaling constitutes a conserved mechanism that modulates locomotion of both prostate and breast cancer cells. In several metastatic breast cancer lines, WSS induces morphological adaptations, increases cellular velocity, and elevates nuclear localization of YAP. Conversely, flow induces cytoplasmic sequestration of YAP and concomitantly slows cell migration in a separate subset of breast cancer lines and an immortalized breast line. Surprisingly, knockdown of YAP did not abrogate response to flow. Instead, YAP and/or YAP/TAZ knockdown increased motility, and YAP constitutive activation reduced motility. Our data demonstrate that WSS regulates YAP but that, unlike prostate cancer cells, other flow‐sensing signaling mechanisms compete with and override YAP activity to drive breast cancer cell taxis. We show that shear stress modifies RhoA GTPase activity differently in positive and negative responders. Our results collectively implicate a force‐responsive program that drives an amoeboid cell state and triggers high levels of RhoA‐ROCK signaling to speed migration of a subset of breast cancer cells.

## 2 MATERIALS AND METHODS

### 2.1 Cell culture and pharmacological reagents

Lines were purchased from American Type Culture Collection (ATCC, Manassas, VA) or DSMZ‐German Collection of Microorganisms and Cell Cultures. Cell line authentication and mycoplasma negativity were confirmed for all cell lines by IDEXX (Westbrook, ME). MCF‐7 breast cancer line was grown in Dulbecco's MEM with 10% fetal bovine serum (FBS, R&D Systems, Minneapolis, MN). The MCF‐10A breast line was grown in MEBM (ATCC, Manassas, VA) with MEB growth supplements. Other human breast cancer lines (EFM‐19, HCC1806, HCC1187, MDA‐MB‐231, and MDA‐MB‐415) were grown in RPMI (Corning Life Sciences, Tewksbury, MA) with 10% FBS. All culture media contained 1% penicillin and streptomycin antibiotics, and cell lines were cultured at 37°C in 5% CO_2_ (v/v). Cells were routinely passaged on tissue culture plastic and transferred into microfluidic channels 20 h prior to application of WSS. The cell line 293FT (Thermo‐Fisher, Waltham, MA) was cultured according to vendor guidelines for virus production and maintenance.

### 2.2 Microfluidic devices and application of wall shear stress

Commercial microfluidic devices (ibidi, Fitchburg, WI) were coated with 50 μg/ml Type I Collagen (Corning, Bedford, MA) at 4°C overnight and breast cancer cells were seeded at a density of one million cells per ml. Due to poor adherence of the HCC1187 breast line, devices used for culture of HCC1187 were coated with 200 μg/ml Type I Collagen to encourage attachment. Following an overnight culture period, unidirectional media flow was introduced through the channel at a constant flow rate using a programmable syringe pump (Harvard Apparatus, Holliston, MA, PhD Ultra). Viscosity of media with 10% FBS at 37°C was approximated as 0.007 dyne s/cm^2^. Based on this viscosity and the dimensions of the culture channels, we applied flow rates of 55 μl/min on ibidi μ‐slides I^0.4^ and 40 μl/min on μ‐slides VI^0.4^, corresponding to 0.05 dyne cm^−2^ WSS. Comparisons were conducted in parallel with static cultures on equivalent microfluidic devices.

### 2.3 Time‐lapse imaging

For quantification of motility, microfluidic channel slides were placed on an inverted microscope (Olympus, Center Valley, PA, IX‐81) and cellular motility data were acquired with phase contrast microscopy using MetaMorph for Olympus software under static or laminar flow conditions. At a given flow rate, successive images across five non‐overlapping fields of view were recorded every 5 min for 6 h in an environmental chamber maintained at 37°C in 5% CO_2_. Cells were tracked in time‐lapse image sequences using the Manual Tracking plug‐in for Fiji (Image J, NIH, Bethesda, MA). The Manual Tracking output included position and velocity measurements for each cell, based on a size of 0.6 µm/pixel. Total distances traveled and average velocities were compiled for each cell from a single position (>100 cells/line) or a minimum of six cells per position at 15 positions (>90–200 cells/line). The HCC1187 cell line was the exception, with 73 cells tracked under WSS and 90 for static.

### 2.4 YAP immunofluorescence

Cells were fixed in 4% paraformaldehyde for 15 min and blocked by 5% bovine serum albumin in PBS‐T (PBS with 0.1% Triton x‐100) for 1 h at room temperature. Cells were treated with mouse anti‐YAP monoclonal antibody (1:100 dilution, Abnova, Walnut, CA, clone 2F12) diluted with 1% bovine serum albumin in PBS‐T at 4**°**C overnight, followed by Alexa 488‐conjugated rabbit anti‐mouse secondary antibody (1:500 dilution, Invitrogen, Waltham, MA, Cat. No. A11059). Counterstaining for each condition was performed with Draq5 (Invitrogen, Waltham, MA). Images were captured by a Leica TCS SP5 confocal microscope (Leica, Wetzlar, Hesse, Germany) with a Leica 63x oil objective lens (NA 1.4) and analyzed with Leica Application Suite software (v. 2.6.3). YAP was manually categorized as primarily nuclear, cytoplasmic, or broadly distributed in both compartments for all cells within the field of view at five positions per condition, and subsequently normalized based upon total number of cells counted using Leica LAS X and ImageJ software packages.

### 2.5 Immunoblotting

Cells were harvested in chilled RIPA Cell Lysis Buffer with EDTA (GenDEPOT, Barker, TX, R4100‐010) with 1% protease and phosphatase inhibitor cocktails (Halt, ThermoFisher, Waltham, MA). After protein determination and normalization between conditions, SDS‐PAGE was performed using Tris‐HCl poured gels. Electrophoretic transfer from gel to nitrocellulose blot was performed with the eBlot L1 Transfer System (GenScript, Piscataway, NJ). Western blotting was performed using antibodies against YAP (Santa Cruz, Dallas, TX, clone H‐9, Cat. No. sc‐271134), phospho‐YAP (Ser127; Cell Signaling Technology, Danvers, MA, Cat. No. 4911), β‐actin (Santa Cruz, Dallas, TX, clone C4, Cat. No. sc‐47778), GAPDH (Cell Signaling Technology, Danvers, MA, 14C10, Rabbit mAb #2118), HA tag (Santa Cruz, Dallas, TX, clone F‐7, Cat. No. sc‐7392), and DYKDDDDK Tag (FLAG; Cell Signaling Technology, Danvers, MA, Cat. No. 2368S). After secondary HRP, Western Sure Chemiluminescent substrate (LI‐COR, Lincoln, NE) was applied. The LI‐COR C‐DiGit chemiluminescent blot scanner was used to scan blots and the Image Studio software (LI‐COR, Lincoln, NE) to quantify band intensities between conditions.

### 2.6 Constructs for ectopic expression and knockdown

The plasmids pCMV‐flag‐YAP1 S127A (Addgene #27370),^25^ pCMV‐flag‐YAP1 5SA/S94A (Addgene #33103),^26^ pcDNA3‐HA‐TAZ (Addgene #32839),^27^ and pcDNA3‐HA‐TAZ S89A (Addgene #32840)^27^ were a gift from Kunliang Guan, and the control vector pEGFP‐N1 was obtained from Clontech. Transfection was performed using 1 µg of plasmid with FuGENE6 (Promega, Madison, WI). pLIPE‐3XHA‐RhoA (V14A) and pLIPE‐3XHA‐RhoA (N19A) retroviral vectors were gifts from Richard Hynes. Other viral vectors were provided and described previously by John Lamar. These included MSCV‐IRES‐Hygro (control vector), MSCV‐flag‐YAP‐IRES‐Hygro, MSCV‐flag‐YAP(S127A)‐IRES‐Hygro, and MSCV‐flag‐YAP(S127A,S381A)‐IRES‐Hygro for YAP overexpression,^22^ and MSCV‐Zsgreen‐2A‐Puro‐shRNA‐FF (control vector), MSCV‐Zsgreen‐2A‐Puro‐shRNA‐hYAP‐1, MSCV‐Zsgreen‐2A‐Puro‐shRNA‐hYAP‐2, and MSCV‐Zsgreen‐2A‐Puro‐shRNA‐hYAP‐7 for shRNA‐based YAP knockdown.^28^

### 2.7 Generation of retrovirus

Retroviruses were produced in HEK‐293FT cells, which were seeded at 200,000 cells per well in a 6‐well plate to obtain near 50% confluency in 2 ml complete media volume. After 18 to 24 h, the cells were transfected with 1 µg of the viral vector (YAP or shRNA YAP), 0.5 µg of the packaging vector plasmid gag/pol (Addgene, Watertown, MA), 0.5 µg of the coat protein plasmid VSV.G (Addgene, Watertown, MA), and 5 µl of the X‐tremeGENE 9 DNA transfection reagent (Roche, San Francisco, CA). This mix was added to 95 µl of Opti‐MEM media (Thermo‐Fisher, Waltham, MA). After 15 min incubation at room temperature, the transfection complex was added dropwise with gentle swirling of the plate into the well (with the same 2‐ml medium). After 24 h of incubation, the medium was aspirated and fresh complete media was added. After another 24 h of incubation, the medium was collected and passed through a 0.45‐µm syringe filter. Supernatant was used immediately or stored at −80°C for later infections.

### 2.8 Retroviral infection

Breast lines were seeded at 50,000 cells per well in a 12‐well tissue culture plate. After 18 h, viral supernatant was diluted 1:1 with fresh complete media and added to the cells. Polybrene Infection/Transfection Reagent (Sigma, St. Louis, MO) was added to the virus at 8 µg/ml. After 24 h, viral supernatant was replaced with fresh complete media containing antibiotic for stable selection (puromycin or hygromycin). After 6–10 days, breast lines were cultured and harvested for WSS experiments.

### 2.9 siRNA‐based gene silencing

SMARTpool siRNAs against YAP and TAZ were from Dharmacon. For siRNA transfection, cells were cultured in standard conditions and transfected using DharmaFECT 1 (Dharmacon). Briefly, cells were plated at 70% confluence and transfected the next day using 25 nM of final concentration of each siRNA. The following day, cells were transferred to channels for WSS application.

### 2.10 Activity assays for RhoA, Rac1, and Cdc42

Levels of GTP‐loaded RhoA, Rac1, and Cdc42 were determined using G‐LISA Small GTPase Activation Assays (Cytoskeleton, Inc., Denver, CO) specific to each small G‐protein, according to manufacturer instructions. Breast lines were analyzed at specific time points up to 60 min for WSS versus static conditions.

### 2.11 RNA sequencing

Cells seeded at a density of two million cells per ml were lysed directly on microfluidic channels in RLT lysis buffer, and total RNA was isolated (RNeasy Micro kit, Qiagen, Germantown, MD), according to the manufacturer's instructions. RNA was measured using a Qubit RNA high‐sensitivity assay and an Agilent Bioanalyzer total RNA 6000 Pico chip. A KAPA mRNA HyperPrep protocol for Illumina platforms was used for poly(A) mRNA capture and construction of reverse‐stranded mRNA‐Seq libraries. Briefly, mRNA was captured using oligo‐dT beads, fragmented with heat and magnesium, and cDNA synthesized according to manufacturer guidelines. Libraries were ligated to KAPA adapter sequences and amplified. cDNA was quality checked on an Agilent Bioanalyzer high‐sensitivity DNA assay chip. Equimolar amounts of each sequencing library were pooled and purified with magnetic KAPA Pure Beads. Pool was sequenced on the Illumina NextSeq 550 using the NextSeq500/550 High Output kit v2.5 (2 × 75 paired end cycles).

### 2.12 Differential gene expression and pathway analysis

Raw reads were checked with FASTQC for quality and trimmed with Trimmomatic prior to mapping using RNA STAR (Galaxy Version 2.7.8a) against the human GRCh38 assembly.^29^, ^30^ Mapping quality was verified by SAMtools idxstats, Picard tools MarkDuplicates (http://broadinstitute.github.io/picard/), and MultiQC.^31^, ^32^ Gene counts were produced with featureCounts assuming reverse strandness.^33^ Differentially expressed genes were identified using DESeq2 and limma‐voom.^34^, ^35^, ^36^ Contrasts used to identify genes contributing to the migration response of breast cancer cells were defined by comparison of the positive responder group's change in gene expression relative to the negative responder groups change (PosWSS‐PosStatic)‐(NegWSS‐NegStatic). Single‐group analyses and data are presented for WSS‐Static contrasts for positive breast, negative breast, and prostate cancer samples. Gene set enrichment analysis was performed in R with EGSEA by computing overlap with MSigDB gene sets, GeneSetDB, and KEGG pathways.^37^, ^38^, ^39^, ^40^, ^41^, ^42^ NCBI's Gene Expression Omnibus (GEO) Accession Number GSE73284 data were used for comparative analyses of prostate and breast cancer signaling using the same limma and EGSEA pipelines, with the exception that prostate cancer was background corrected and control probes removed in a workflow appropriate for Illumina beadchips.^43^, ^44^, ^45^ Bioconductor packages for R were used to generate plots with EnhancedVolcano (https://github.com/kevinblighe/EnhancedVolcano) and KEGG pathway maps with Pathview.^46^ The RNA‐seq data for breast cancer have been deposited in NCBI's GEO repository and are available through the GEO repository under series Accession Number GSE191142.

### 2.13 RT‐qPCR gene expression analysis

Reverse transcription of RNA was performed using the High‐Capacity cDNA Reverse Transcription Kit (Applied Biosystems, Waltham, MA), according to the manufacturer's instructions. Real‐time TaqMan PCR (Applied Biosystems, Waltham, MA) was performed in 10 μl reactions with primers provided by Applied Biosystems and TaqMan Universal PCR Master Mix, no AmpErase UNG, according to the manufacturer's instructions. For calculation of fold change, cycle thresholds (Ct) were determined using Roche LightCycler 480 software (Roche, San Francisco, CA), and mRNA expression was normalized to GAPDH transcript and the static control sample.

### 2.14 Statistical analysis

All data were analyzed with SigmaPlot 12.5 (Systat Software Inc., San Jose, CA) for statistical significance and are reported as mean ± standard deviation using GraphPad Prism 9.0 (San Diego, CA). Parametric tests were used only if data met assumptions of normality and homoscedasticity. Differences in migration were analyzed with the two‐tailed unpaired *t*‐test when experimental design included only two groups or by one‐way ANOVA with Holm‐Sidak or Dunn's post‐hoc comparisons for experiments that included three or more groups. Two‐way ANOVA was used for the analysis of treatment groups with two variables, such as force application and genetic knockdown. Band intensities of Western blots with three or more treatment groups were analyzed by one‐way ANOVA with Holm‐Sidak. Significance levels of *p* < 0.05, 0.01, or 0.001 are denoted in graphs by single, double, or triple asterisks, respectively. Representative results from at least three independent biological replicates are shown unless stated otherwise.

## 3 RESULTS

### 3.1 Intrinsic cell properties modulate response to force

**FIGURE 1:**
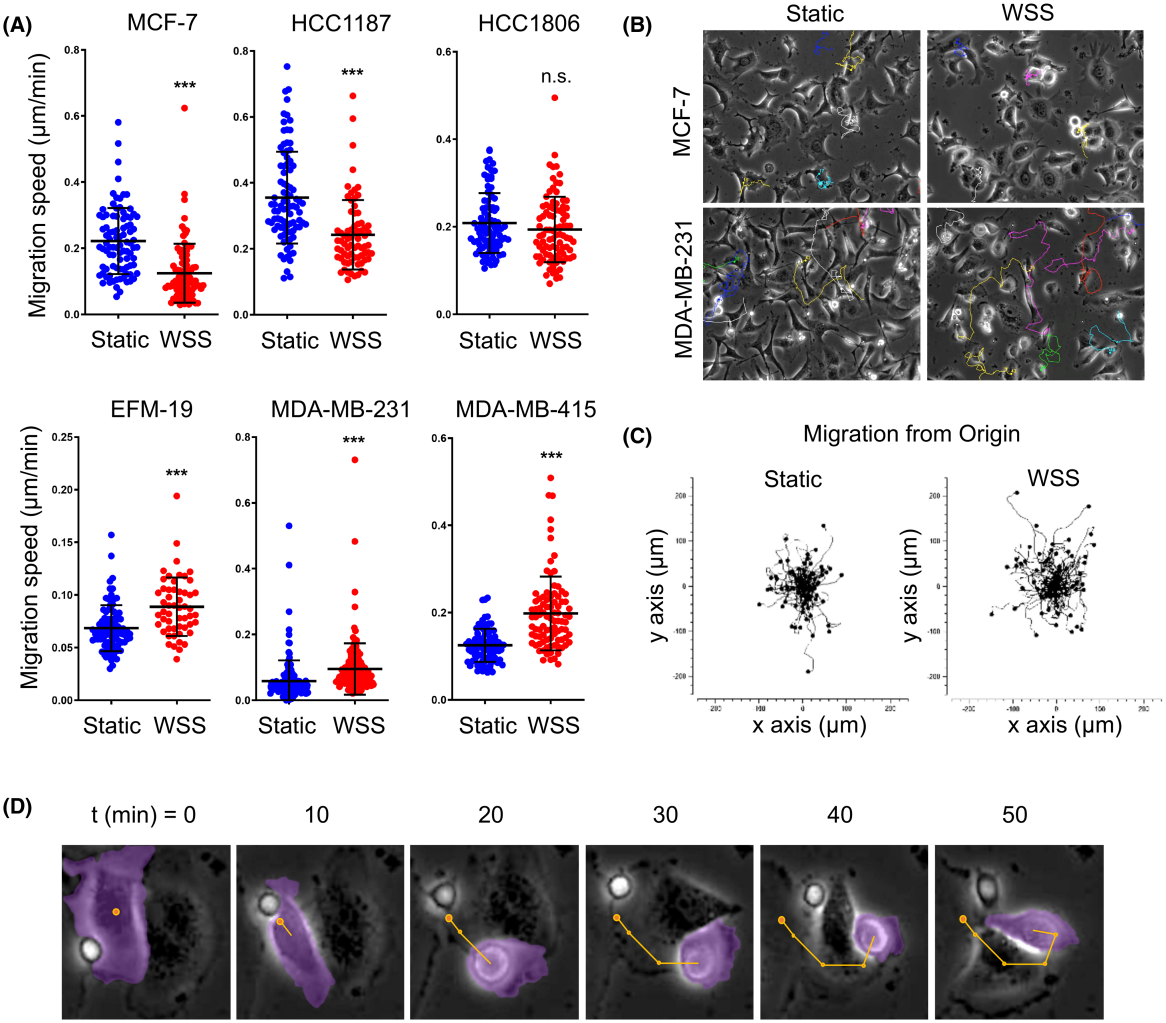
Flow can promote or inhibit motility in breast cancer cell lines. (A) Tracking analysis shows that cellular velocity is altered by flow in various breast cancer cell lines. Wall shear stress (WSS) exposure significantly increases motility in EFM‐19, MDA‐MB‐231, and MDA‐MB‐415 cells; whereas, WSS inhibits migration in MCF‐7 and HCC1187 lines. HCC1806 exhibits no detectable change in motility. Results are expressed as mean ± SD; *n* ≥ 70 individual cells for all lines (unpaired two‐tailed *t*‐test, ***p < 0.001). (B, C) Still photos from time‐lapse movie files and representative cumulative plots show that cell migration radiates from the origin of each cell's starting location with no overt directionality. (D) Magnified view of single MDA‐MB‐231 cell undergoing a shift toward amoeboid morphology within minutes of initiation of WSS. Purple pseudocolor encompasses cell body and membrane projections. Yellow dot and lines mark starting location and path of locomotion

Tumor invasion mechanisms are diverse, and the multistage process of metastasis requires adaptability of cells to microenvironments within primary tumors, the ECM, the blood or lymphatic circulation, and finally the secondary metastatic niche. Cells exhibit great plasticity in locomotion, though three of the chief modes of migration are generally classified as mesenchymal, amoeboid, and collective. At the core of a neoplastic tumor, cells typically use an elongated mesenchymal style, while cells escaping the tumor boundary enter the ECM using rounded amoeboid motility.^47^ Breast cancers arise from discrete origins and harbor distinct genetic mutations, thus could have differing responses to microenvironmental cues and biophysical forces. To address the complexity and variation in breast cancer subtypes, we examined motility response to flow in the MCF‐10A non‐tumorigenic epithelial cell line and six breast cancer cell lines with varying levels of metastatic potential, including EFM‐19, HCC1187, HCC1806, MCF‐7, MDA‐MB‐231, and MDA‐MB‐415 (Table 1). To mimic lymph flow, 0.05 dyne cm^−2^ WSS corresponding to the velocity of fluid flow in the initial lymphatics and interstitium was applied to cells adherent within microfluidics.^48^, ^49^ We monitored taxis by time‐lapse imaging for 6 h, followed by manual tracking of randomly selected cells at five positions within microfluidic channels. Motility observed in these assays represented single cell migration, as quantification of intra‐cluster motility would have required more sophisticated labeling of individual cells. Thus, collective or intraspheroid‐type motility within duct‐like clusters of cells was not measured. For example, HCC1187 cells exhibited clustering behavior that made single cell tracing difficult and many “leading” cells carried clumps of cells that floated above the culture surface. Other lines migrated singly, and, upon exposure to WSS, EFM‐19, MDA‐MB‐231, and MDA‐MB‐415 significantly increased velocity (Figure 1A; unpaired *t*‐test, ***p < 0.0001). In contrast, the immortalized breast line MCF‐10A and two cancer lines, MCF‐7 and the clumping cell line HCC1187, significantly decreased motility (Figure 1A; Figure S1A; unpaired *t*‐test, ***p < 0.0001). Cell motility in the highly metastatic HCC1806 line was not significantly altered. Migration did not appear to be directional relative to flow in any line (Figure 1B and C; Movie [Link], [Link], [Link], [Link]). Table 2 summarizes velocity and total distance traveled. A striking similarity across lines that increased taxis was induction of a rounded cell body with alternating prominence of leading edge and posterior uropod (Figure 1D). This amoeboid morphology was adopted in a large fraction of cells within 20 min of WSS initiation and was sustained by 40 min, albeit dynamically, throughout the duration of the exposure to flow. Amoeboid cancer cells are enriched in the invasive fronts of primary tumors and in secondary lesions.^50^, ^51^, ^52^, ^53^, ^54^ Critically, amoeboid locomotion facilitates more rapid exploration of the environment and is known to amplify aggressive and metastatic behavior of epithelial tumors, such as breast cancer, prostate cancer, and hepatocellular carcinoma.^55^, ^56^, ^57^, ^58^, ^59^ Our data are consistent with the notion that differential response to extrinsic force is both determined by intrinsic properties of cancer cells and that differences in force‐sensing machinery can be profound within and between cancer types.

**TABLE 1:** Breast cell line properties

Cell line	Catalog no. (vendor)	Disease	Subtype^97^, ^98^	Derived from	PTEN status^99^	TP53 status^98^, ^100^	HER2	ER	PR^98^
EFM‐19	ACC 231 (DSMZ)	Invasive ductal carcinoma	Luminal	Pleural effusion	WT	H193R	−	+	−
HCC1187	CRL‐2322 (ATCC)	Ductal carcinoma	Basal A	Tumor	WT	G108del, +	−	−	−
HCC1806	CRL‐2335 (ATCC)	Squamous carcinoma	Basal A	Tumor	WT	T256fs*90	−	−	−
MCF‐10A	CRL‐10317 (ATCC)	Fibrocystic disease	Basal, nonmalignant	Breast	WT	WT	−	−	−
MCF‐7	HTB‐22 (ATCC)	Metastatic adenocarcinoma	Luminal	Pleural effusion	WT	WT	−	+	+
MDA‐MB‐231	HTB‐26 (ATCC)	Metastatic adenocarcinoma	Basal B, post‐EMT	Pleural effusion	WT	R280K, +	−	−	−
MDA‐MB‐415	HTB‐128 (ATCC)	Adenocarcinoma	Luminal	Pleural effusion	C136Y, ±	Y236C, ±	−	+	−

**TABLE 2:** Breast line motility and YAP localization

Cell line	No. cells tracked		Velocity ± SD (µm/min)		Distance ± SD (µm)		Change with WSS	
	Static	Wall shear stress (WSS)	Static	WSS	Static	WSS	Motility	Nuclear YAP
EFM‐19	90	90	0.07 ± 0.02	0.09 ± 0.03	24.7 ± 7.9	31.8 ± 9.7	+	+
HCC1187	90	73	0.36 ± 0.14	0.24 ± 0.11	127.8 ± 50.2	87.3 ± 38.0	−	+
HCC1806	269	261	0.21 ± 0.01	0.19 ± 0.01	75.1 ± 2.6	69.7 ± 2.8	n.s.	+
MCF‐10A	186	156	0.72 ± 0.25	0.56 ± 0.19	265.0 ± 68.7	221.0 ± 51.4	−	−
MCF‐7	90	90	0.22 ± 0.10	0.12 ± 0.09	79.9 ± 35.9	43.9 ± 32.1	−	−
MDA‐MB‐231	439	289	0.06 ± 0.00	0.09 ± 0.00	22.4 ± 1.1	32.5 ± 1.5	+	+
MDA‐MB‐415	90	90	0.13 ± 0.04	0.20 ± 0.09	45.0 ± 13.7	70.5 ± 30.5	+	+

### 3.2 Motility behavior segregates with cell adhesion and HIPPO pathway regulation

**FIGURE 2:**
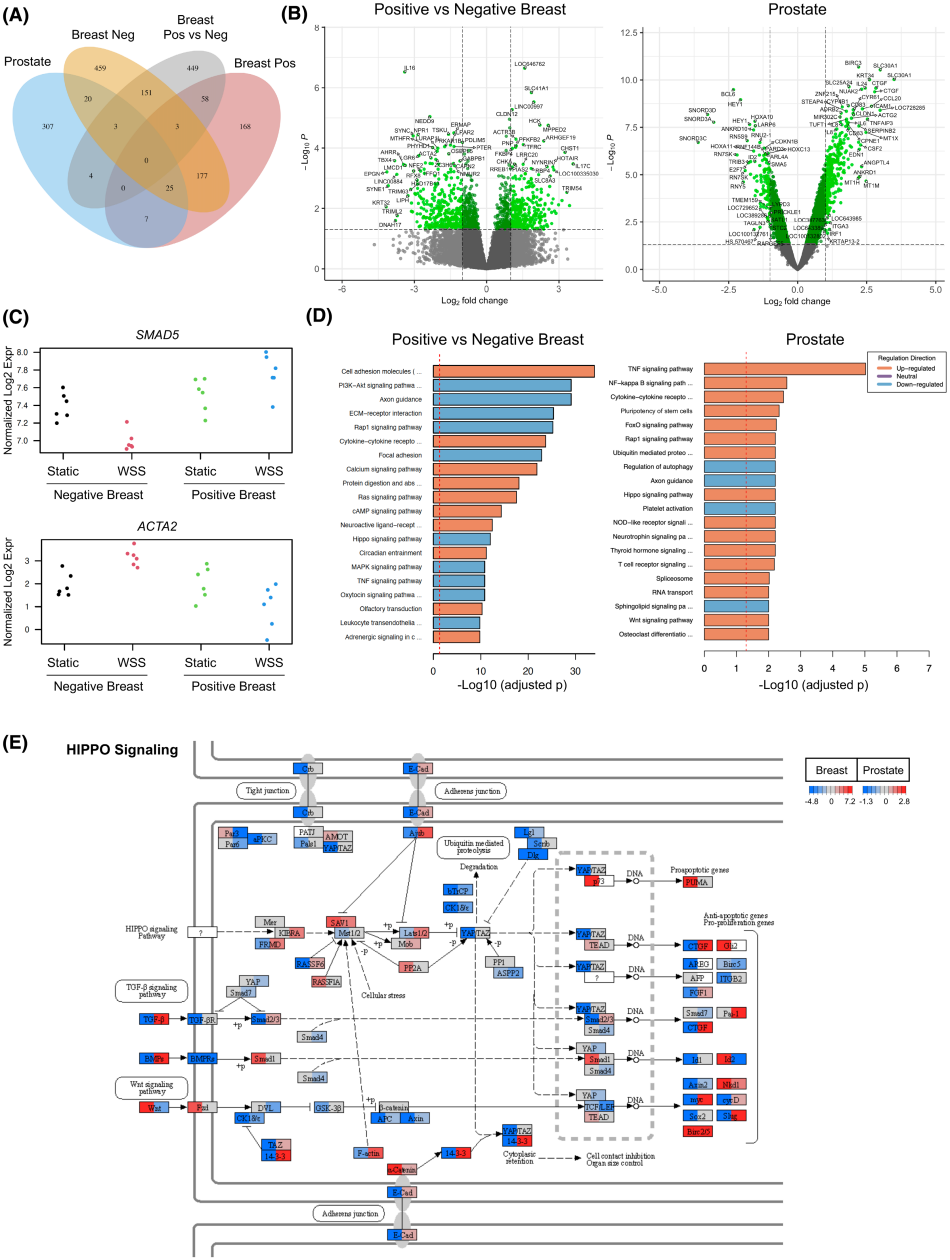
Positive and negative motility response defines transcriptionally distinct subsets of cancer cells. (A) Venn diagram illustrates overlap and uniqueness of gene expression signatures induced by lymphatic intensities of wall shear stress (WSS) in PC3 prostate cancer cells and two subsets of breast cancer cells defined by increased (positive) or decreased (negative) migration response under flow. The positive versus negative comparison contrasts the two breast cancer subsets. (B) Differentially expressed genes are depicted by volcano plot with cutoffs demarcated by dashed lines and color at *p*‐value < 0.05 and −1 < log2 fold change < 1. (C) Transcripts of genes representative of those regulated in distinct directions in the breast cancer subsets. (D) GSEA of top 20 KEGG pathways show broad changes in adhesion, cytokine signaling, and cytoskeleton. Contrast reflects change relative to static conditions such that orange bars indicate pathways upregulated in positive breast cancer responders exposed to flow relative to negative responders exposed to flow. Blue bars depict pathways predicted to be more highly downregulated in positive responders relative to negative responders. Prostate cancer pathways reflect WSS versus static conditions. (E) Expression of HIPPO pathway components are shown to indicate fold change in positive versus negative breast cancer comparison in the left half of each split node and prostate on the right of each node. Color scale legends indicate fold change in gene expression following 6 h of WSS in breast or prostate

We evaluated breast and prostate cancer transcriptomes for clues to the signaling that dictates whether cells increase (positive) or decrease (negative) cell movement under fluid shear stress. Breast cancer subsets subjected to bulk RNA‐seq were grouped for differential gene expression analysis by positive or negative response to flow (Dataset 1). Prostate cancer cells were included in parallel analyses to assess the degree of conservation of signaling in a cancer type known to move more rapidly under flow (Dataset 1). Genes regulated by WSS differed between breast subsets and between breast and prostate (Figure 2A; −1 < log2 fold change < 1, adjusted *p*‐value < 0.05). Twenty‐five genes were shared among all three groups, suggesting that these could be highly conserved WSS‐responsive genes. Seven genes were shared between prostate and positive breast cancer responders (*TRNT1*, *NUFIP1*, *TDG*, *HSPA8*, *OTUD6B*, *RBM12*, and *DENND3*). Rather than identify all genes that are up‐ or down‐regulated with flow, we designed an analysis pipeline capable of retrieving gene candidates that correlate with motility response (controlled by internal normalization within each cell line). This method also reduced background introduced by intrinsic heterogeneity of breast cancer cell lines. Thus, to specifically address the signaling responsible for motility behavior, we conducted analyses to examine which genes were most differentially expressed between positive and negative breast cancer responders (Figure 2A and B). Particularly interesting were those genes upregulated in positive responders but downregulated in negative responders, exemplified by *SMAD5*, and the reverse pattern, as observed with *ACTA2* (Figure 2C). Seven of the top genes from this comparison were also differentially expressed in prostate, three shared with negative responders (*CHAC1*, *LIF*, and *GABPB1*) and four not shared with either breast subset (*TRIB3*, *NT5DC3*, *BACH2*, and *PDLIM5*).

We explored the relevance of these expression changes to signaling by gene set enrichment analysis (GSEA) with a collection of databases encompassing KEGG, MSigDB, and GeneSetDB (Figures S2 and S3; Dataset 2, 3, 4, and 5). Cell adhesion molecules, PI3K‐Akt signaling, axon guidance, ECM–receptor interaction, Rap1 signaling, cytokine–cytokine receptors, and focal adhesion were among the top KEGG pathways that differed between positive versus negative responding breast cancer cells (Figure 2D). The observed downregulation of adhesion molecules, integrins, and ECM interactions in positive responders is wholly consistent with adoption of an amoeboid mode of motility (Figure S2B, C). Indeed, prior reports indicate that some carcinoma cells move at accelerated rates with an amoeboid morphology (up to 4 μm/min) in vivo and that this motility style is largely independent of cell–ECM contact and proteolytic degradation of ECM by metalloproteases.^60^, ^61^ Strikingly, some of the pathways differentially expressed between positive versus negative responders, including NF‐κB, have been implicated in the epithelial to amoeboid transition (EAT)^53^ (Figure S2C). Not surprisingly, HIPPO signaling was identified in both prostate and breast as the top 10th and 13th enriched KEGG pathway, respectively. Unexpected was the predicted direction of regulation, which was upregulated in prostate and downregulated in breast. In other words, the positive responding breast lines appeared to downregulate HIPPO signaling relative to the negative responders, and this was inconsistent with the pattern observed and expected for the PC3 prostate cancer cells, which are known to positively respond to force and activate YAP, a negative effector of HIPPO signaling. We observed prominent induction of several classic YAP/TAZ target genes in prostate cancer, including *CTGF*, *CYR61*, and *ANKRD1* (Figure 2B), yet this enrichment of YAP/TAZ downstream targets was less striking in the comparisons of breast subsets (Figure 2B; Figure S2). Closer examination of HIPPO signaling ligands and effectors revealed consistent differential regulation in breast and prostate (Figure 2E). Genes encoding mediators of cytoplasmic retention of YAP such as 14‐3‐3 and Lats1/2 were downregulated by flow in positive responding breast cells and upregulated in prostate cancer cells. TGF‐β and BMPs were down in positive breast lines and up in prostate. Given the enrichment of differentially expressed HIPPO signaling genes and prior evidence for a role of YAP/TAZ in mediating force‐induced motility, we concluded that YAP could play a role in determining migration behavior of breast cancer cells.

### 3.3 Cell motility correlates with YAP subcellular localization but not activation of YAP/TAZ target genes

**FIGURE 3:**
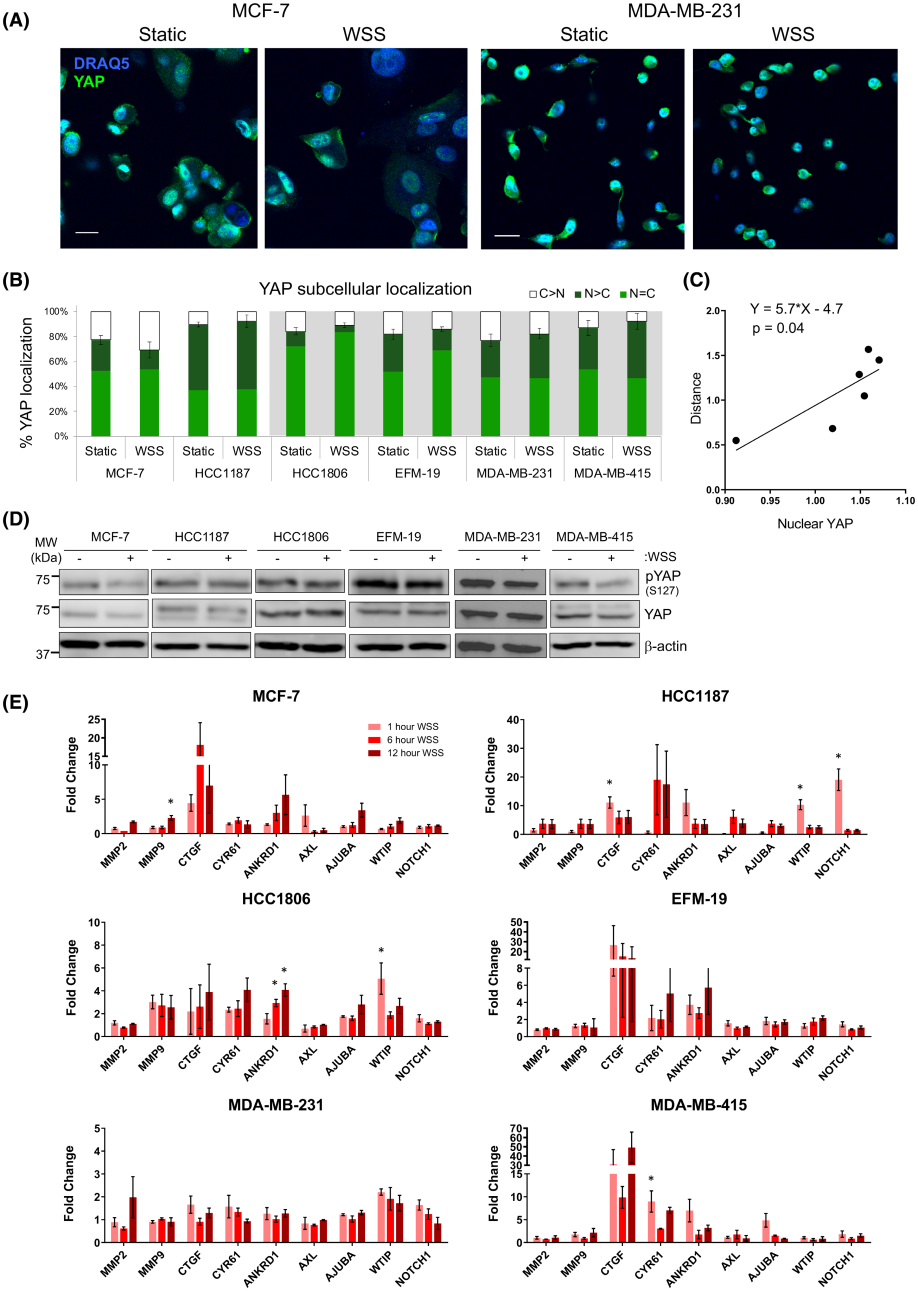
YAP nuclear localization positively correlates with motility. (A) Representative images of immunofluorescent staining show altered YAP subcellular localization upon exposure to flow. Scale bar represents 25 μm. (B) Quantification demonstrates that the fraction of cells containing YAP in the nucleus decreases in MCF‐7 but increases in other lines, such as MDA‐MB‐231 and MDA‐MB‐415. Grey background demarcates cancer lines with increased frequency of cells containing nuclear YAP under flow (N > C and N = C). Error bars represent SEM. (C) Fold change in distance traveled and nuclear YAP are significantly correlated across all cell lines (Linear regression, *p* = 0.04). (D) Dephosphorylation of YAP S127 is triggered by flow in MCF‐7, EFM‐19, MDA‐MB‐231, and MDA‐MB‐415 cell lines. (E) Transcript levels of seven genes classically used as readouts of YAP and/or TAZ activity, such as *CTGF*, were measured at 1, 6, or 12 h after initiation of wall shear stress. Expression of these genes was stimulated by flow to varying extents in all breast lines tested (one‐way ANOVA, *p < 0.05 relative to static control). Metalloprotease genes *MMP2* and *MMP9* were also evaluated as indicators of invasive potential. Error bars represent SEM

In prostate cancer cells, YAP translocates to the nucleus on exposure to flow and is required for flow‐enhanced motility.^18^ Dephosphorylation of the YAP serine residue at position 127 (S127) enables transit to the nucleus and permits transactivation of target genes that regulate cell movement, survival, and proliferation. YAP activity as a transcriptional cofactor on gene promoters is critical to its ability to drive taxis. To evaluate YAP regulation in response to flow in breast cancer, cell lines were exposed to static or WSS conditions for 6 h, fixed, and immunostained for detection of YAP. WSS promoted sequestration of YAP in the cytoplasm of the nontumorigenic MCF‐10A cell line (Figure S1B, C) and in MCF‐7, two cell lines that slowed migration in response to WSS (Figure 3A and B). Nuclear localization increased with WSS in HCC1187, HCC1806, EFM‐19, MDA‐MB‐231, and MDA‐MB‐415 cell lines (Figure 3A and B; Figure S4A). Increased velocity in response to flow positively correlated with nuclear localization of YAP (Figure 3C; Table 2; Figure S4B). The outlier to this pattern was HCC1187, which had strong tendency to form cell clusters in lieu of migrating singly. S127 dephosphorylation was not a reliable indicator of YAP subcellular localization in breast lines nor could it predict motility response (Figure 3D; Figure S1D, S4B). This result is, however, consistent with prior reports suggesting that phosphorylation at S127 alone might not be sufficient to dictate YAP localization.^25^, ^62^, ^63^, ^64^ These data suggested that fluid force directs both YAP subcellular localization and taxis. We, therefore, hypothesized that flow‐induced transit of YAP to the nucleus could regulate genes that drive motility response in breast cancer cells.

We evaluated the functional relevance of YAP nuclear localization by analysis of mRNA levels of classical YAP/TAZ target genes, along with metalloprotease and Notch transcripts. No clear link between motility behavior and abundance of target gene transcripts could be established (Figure 3E; Figure S1E). For example, *CTGF* transcripts were elevated 20‐ to 50‐fold in both negative and positive flow‐responding cell lines, including MCF‐7 (negative) and EFM‐19 and MDA‐MB‐415 (positive). In contrast, increase in *CTGF* was not seen in MDA‐MB‐231 (positive responder). *CYR61* and *ANKRD1* were more modestly induced and had no clear correlation with velocity of taxis. Given that *CTGF*, *CYR61*, and *ANKRD1* have also been shown to be regulated by the actin‐sensitive MRTF‐SRF complex downstream of Rho‐GTPase signals,^65^ their transcript abundance could instead reflect altered G actin concentration. Flow was also a poor inducer of the metalloprotease genes *MMP2* and *MMP9* in the breast cancer cell lines evaluated, suggesting that WSS of low magnitude likely plays little role in stimulating breast cancer invasion into extracellular matrices. These data were consistent with the amoeboid motility observed in positive responding breast lines, as proteases are not required for this style of motility because cells squeeze through gaps in the ECM instead of degrading it.^66^

### 3.4 YAP is neither necessary nor sufficient to drive breast cancer cell migration

**FIGURE 4:**
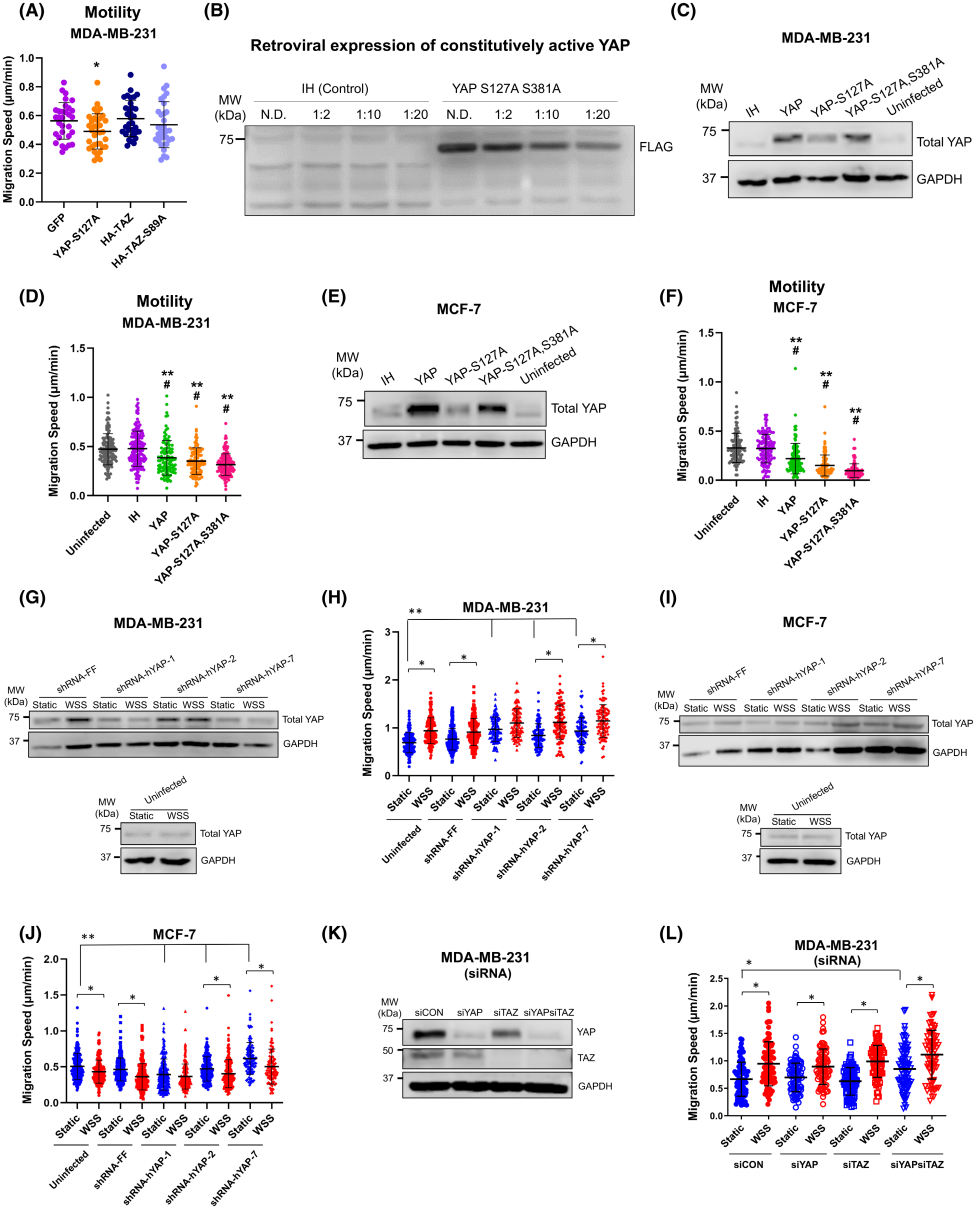
YAP constitutive activation inhibits whereas knockdown promotes breast cancer cell migration. (A) Ectopic expression of constitutively active forms of YAP and TAZ by transient transfection of plasmids used in our prior study of prostate cancer failed to induce motility under static conditions. Instead, constitutively active YAP S127A suppressed taxis (one‐way ANOVA Dunn's, *p < 0.05). pEGFP‐N1 served as the control vector. (B) As a complementary approach, retrovirus was used to express YAP S127A,S381A in MDA‐MB‐231. Blot shows that FLAG tag is detectable even when virus is diluted 1:20 during infection. The MSCV‐IRES‐Hygro control vector is demarcated as IH. (C, D) Introduction of constitutively active forms of YAP by retrovirus, including S127A and S127A,S381A mutants, were confirmed to result in elevated total YAP protein levels and suppressed migration speed in MDA‐MB‐231 cells (one‐way ANOVA, **p < 0.01 relative to uninfected control, #*p* < 0.05 relative to IH control). (E, F) Expression of constitutively active YAP was also successful in MCF‐7 cells and similarly reduced migration as opposed to increasing movement (one‐way ANOVA, **p < 0.01 relative to uninfected control, #*p* < 0.05 relative to IH empty vector control). (G, H) Knockdown of YAP by three independent shRNAs did not profoundly alter motility response to WSS by MDA‐MB‐231. Instead, YAP depletion increased cellular velocity under static conditions, along with a proportionate increase under WSS (two‐way ANOVA, *p < 0.05, **p < 0.01). MSCV‐Zsgreen‐2A‐Puro‐shRNA‐FF served as the control for retroviral knockdown. (I, J) MCF‐7 also continued to respond to WSS with reduced motility with or without YAP knockdown (two‐way ANOVA, *p < 0.05, **p < 0.01). (K) siRNAs were used as independent validation to knockdown YAP and TAZ singly and in combination. (L) siRNA‐based YAP or TAZ knockdown alone did not produce amplified migration speeds, but YAP‐TAZ combination knockdown increased motility (two‐way ANOVA, *p < 0.05). Error bars on motility plots represent SD

To rigorously test for a causal relationship between YAP nuclear localization and motility, we expressed constitutively active forms of YAP. We first examined MDA‐MB‐231, which we had found to positively respond to flow by enrichment of YAP in the nucleus and elevated motility. Unexpectedly, constitutively active YAP‐S127A failed to enhance motility in MDA‐MB‐231 (Figure 4A). Instead, YAP‐S127A suppressed migration. These data contrasted with the accelerated taxis observed previously after transfection of the same YAP‐S127A construct in prostate cancer cells.^18^ The failure of wild‐type TAZ and constitutively active TAZ‐S89A to increase locomotion was less surprising, as we have shown that TAZ primarily stimulates proliferation and does not impact motility in prostate cancer^67^ (Figure 4A). A caveat of these transient transfections was that no selection was possible to ensure uniform expression of the insert. We then used a complementary retroviral approach to express active YAP with hygromycin‐based positive selection. We infected cells with YAP, YAP‐S127A, or YAP‐S127A,S381A virus and confirmed by FLAG tag that infection was effective even when virus was diluted 1:20 (Figure 4B). We then measured total YAP expression relative to uninfected and control vector‐infected cells in samples treated with undiluted virus. All YAP constructs produced an increase in total YAP protein (Figure 4C; Figure S5A). MDA‐MB‐231 cells that ectopically expressed YAP and active forms of YAP moved more slowly than the control cells (Figure 4D). This was inconsistent with our hypothesis that YAP could promote motility; therefore, we also evaluated MCF‐7 cells, a line which negatively responded to flow by cytoplasmic sequestration of YAP and reduced taxis. Cellular velocity of MCF‐7 was also decreased by ectopic expression of YAP and activated YAP mutants (Figure 4E and F; Figure S5A). These data suggested that YAP might play a negative role in limiting cell movement, so we designed complementary loss‐of‐function experiments to test the requirement for YAP in mediating motility in static conditions and in response to flow.

The MDA‐MB‐231 positive responder to flow was infected with three independent shRNAs against YAP and selected with puromycin. Cells were cultured under static or WSS conditions, and knockdown of YAP was confirmed by Western blot (Figure 4G; Figure S5B). YAP knockdown accelerated migration, further corroborating the data from overexpression studies showing that YAP negatively regulates taxis in MDA‐MB‐231 cells. MDA‐MB‐231 velocity was also elevated by flow regardless of YAP depletion (Figure 4H). Identical analyses were performed with MCF‐7, which further confirmed that YAP does not drive motility response to WSS (Figure 4I and J; Figure S5B). Because YAP and TAZ regulate the same set of target genes and are known to display redundant activities in MDA‐MB‐231 cells, we examined the effects of knockdown of both YAP and TAZ (Figure 4K and L; Figure S5C). Knockdown of TAZ singly did not alter cellular velocity; however, depletion of YAP and TAZ together produced an overall increase in locomotion. Consistent with the observation that YAP knockdown by shRNA enhanced motility, these experiments further support that YAP/TAZ play an inhibitory role in MDA‐MB‐231 migration. Effects of WSS and YAP/TAZ knockdown were additive, leading to even higher velocities than cells treated with either WSS or knockdown alone. Together, these findings suggest that flow‐regulated taxis occurs irrespective of YAP/TAZ activity, pointing to another flow‐sensing mechanism that adapts motility behavior.

### 3.5 RhoA‐ROCK regulates wall shear stress–induced cellular motility

Five of the top KEGG pathways in the comparison of breast subsets pointed to Rho family GTPases in modification of the actin cytoskeleton and cell movement. Rho controls stress fibers and focal adhesion formation, and Rac and Cdc42 regulate membrane ruffling and filopodium formation, respectively.^68^ Regulation of Rho activity includes input from various signaling pathways and extracellular stimuli. A key source of signaling originates at the plasma membrane, where receptors and molecules that mediate interaction with the ECM and cell–cell contact, such as E‐cadherin, were found to be profoundly different in positive versus negative responders (Figure 2D; Figure S2C; Figure S6). Positive responders overall expressed lower levels of transcripts encoding integrins and adhesion proteins deposited in the ECM, including collagens and laminins (Figure S6A, B; Dataset 1, 4). In contrast, adhesion and ECM interactions were less prominent in prostate cancer cells (Figure 2D; Figure S3).

Receptors at the plasma membrane are essential for regulation of other molecules that mediate activation state of the Rho family members via modulation of their binding to GTP and GTPase activity. The exchange of GDP to GTP is catalyzed by guanine nucleotide exchange factors (GEFs), which act downstream of growth factor receptors, integrins, cytokine receptors, and cadherins.^69^, ^70^ Inactivation of Rho GTPases by weak intrinsic hydrolysis of GTP to GDP requires GTPase activating enzymes (GAPs).^69^, ^71^ Rho and Rac can also use these accessory proteins to engage in reciprocal regulation. Interestingly, amoeboid signaling has been shown to shift cells away from mesenchymal movement, primarily through inhibition of Rac1 by ARHGAP22.^68^ Positive responding breast cancer cells upregulated *ARHGAP22* by log2 fold change of 1.9, suggestive of Rac1 negative regulation. Positive responders also expressed lower levels of the gene that encodes p190RhoGAP, *ARHGAP35*, with a log2 fold change of −0.86. Integrin engagement and subsequent Rac activation has been shown to stimulate the activity of p190RhoGAP, thus downregulating Rho activity by promoting its phosphorylation. In other signaling that balances the pendulum of Rho‐Rac activity, tyrosine kinases, and G‐protein‐coupled receptors (GPCRs) required for Rac activity depend on phosphoinositol3‐kinase (PI3K) activity,^72^ the topmost pathway predicted to be downregulated in positive responders, supporting the notion that Rac1 activity could be downregulated and RhoA upregulated by flow in positive responders. Finally, positive responders also downregulate *SMURF1* (log2 fold change = −0.60), the gene encoding E3‐ubiquitin ligase that targets RhoA for degradation.^73^

**FIGURE 5:**
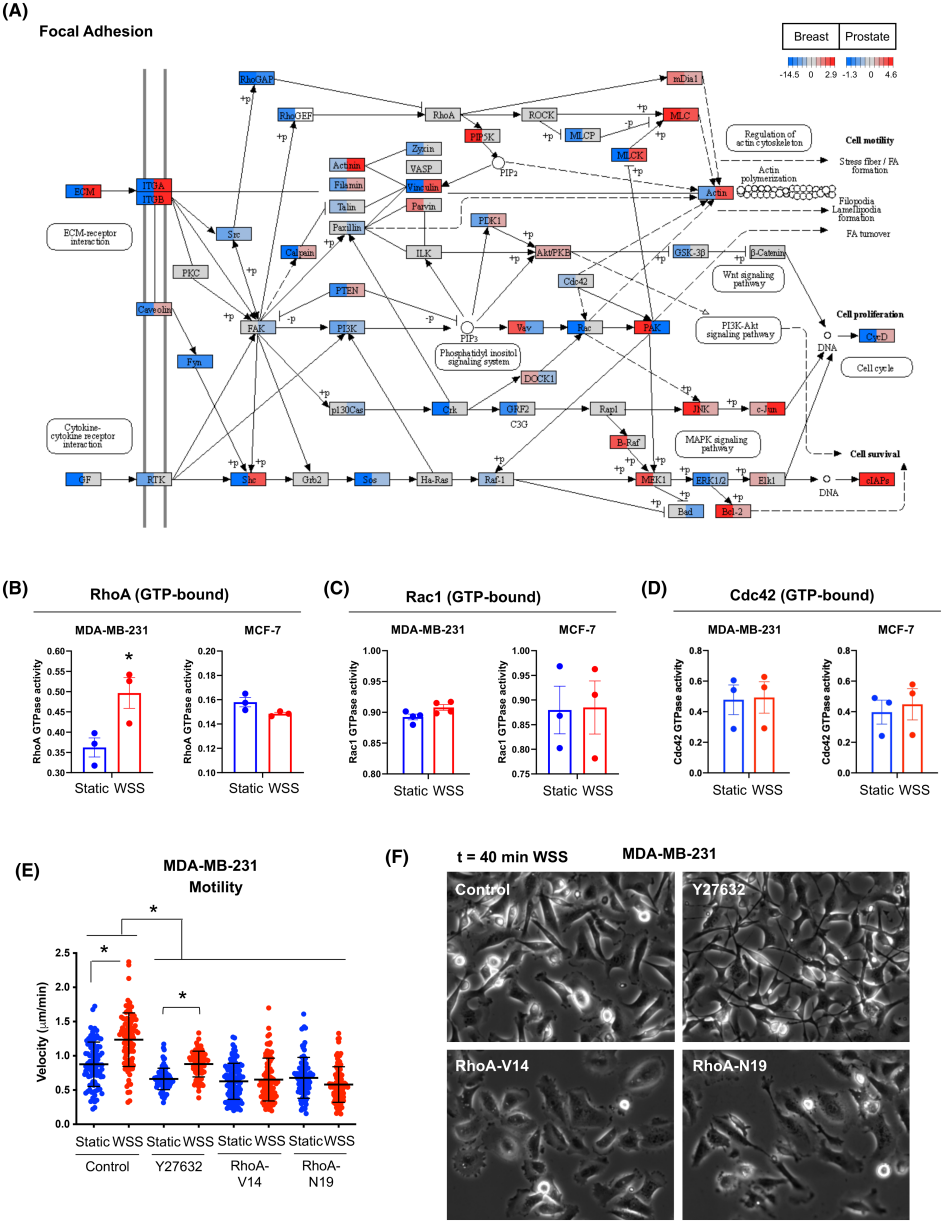
RhoA‐ROCK activity determines motility response to flow. (A) Focal adhesion was identified as a top KEGG pathway differentially regulated between positive and negative breast cancer subsets. Fold change in pathway signaling effectors is depicted for positive versus negative breast cancer contrast in the left half of split nodes and for prostate on the right of the node. Color scale legends indicate fold change after 6 h of wall shear stress (WSS). (B) Flow significantly elevated GTP‐bound (active) RhoA levels 60 min after initiation of WSS in MDA‐MB‐231 (unpaired *t*‐test, *p < 0.05). RhoA GTPase activity was unchanged in MCF‐7. Error bars represent SEM. (C) Rac1 activity was not significantly changed in either MDA‐MB‐231 or MCF‐7. (D) Cdc42 activity was unchanged in response to flow in both cell lines. (E, F) WSS‐induced motility in MDA‐MB‐231 is suppressed by ROCK inhibitor Y27632 (10 µm) and disruption of RhoA activity by dominant negative or constitutively active forms of RhoA (two‐way ANOVA, *p < 0.05). Error bars represent SD

Downstream of Rho, ROCK can directly phosphorylate myosin light chain 2 (MLC2) or indirectly decrease myosin phosphatase (MYPT) activity, thus increasing MLC2 phosphorylation.^74^ The amount of phosphorylated MLC2 controls the myosin II motor protein that together with actin generates intracellular mechanical force important for migration. ROCK appears to be essential for MLC phosphorylation associated with actin filaments in the cell body, whereas MLCK is required for cortical actin at the cell periphery.^75^ This allows the cell to separately control cortical actin dynamics from contractions in the inner cell body and is consistent with our observation that positive responders exhibit upregulated MLC but less MLCK relative to negative responders (Figure 5A). ROCK can also activate LIMK, which phosphorylates and inactivates cofilin, promoting F‐actin stabilization.^76^ Focal adhesion signaling collectively integrates these inputs to regulate actin polymerization and remodeling of cytoskeletal features critical for the process of taxis, including stress fibers, filopodia, lamellipodia, and focal adhesions (Figure 5A). We previously showed that mechanotransduction downstream of flow in prostate cancer cells includes the activation of YAP by ROCK, LIMK, and cofilin.^18^ Given the well‐established roles of the Rho family of GTPases in cytoskeletal organization and migration, as well as their sensitivity to mechanical and chemical stimuli, we hypothesized that breast cancer cells regulate Rho in response to flow. We evaluated the kinetics of RhoA, Rac1, and Cdc42 GTPase status in a positive responder to flow (MDA‐MB‐231) and a negative responder to flow (MCF‐7). Within 30 min of initiation of WSS, active RhoA GTPase (with bound GTP) began to increase, and by 1 h, active RhoA GTPase was significantly elevated (Figure 5B). Timing of RhoA activation was consistent with observations of morphological adaptation to an amoeboid shape upon initiation of flow (Figure 1D). Conversely, MCF‐7 cells did not upregulate RhoA activity in the presence of mechanical force, but rather experienced a slight, albeit non‐significant, drop in GTP‐bound RhoA levels (Figure 5B). Together with the observation that myosin light chain transcripts were elevated (Figure 5A; Dataset 4; *MYL10*, log2 fold change = 1.3, *p *= 0.03), amplified RhoA activity is consistent with a body of evidence that indicate amoeboid behavior is sustained by high levels of Rho‐ROCK‐driven myosin II and short‐lived cell adhesions.^77^ Other Rho family members, Rac1 and Cdc42, were not significantly changed in either cell line (Figure 5C and D). ROCK is a direct downstream target of RhoA but not Rac1 or Cdc42. Thus, we examined the effects of constitutively active RhoA (RhoA‐V14), dominant negative RhoA (RhoA‐N19), and ROCK inhibition in the positive responding cell line, MDA‐MB‐231. ROCK inhibitor, Y27632, or perturbation of RhoA activity culminated in suppressed response to flow, both in morphological adaptation and cell migration (Figure 5E and F). Disruption of coordinated RhoA activity under flow prevented formation of amoeboid morphology and instead resulted in phenotypes ranging from spindle‐shaped to flattened cell bodies (Figure 5F). In summary, our data demonstrate that force‐directed trafficking of YAP does not dictate migration behavior in breast cancer cells. Other inputs critical to mechanosensing such as cell–cell adhesion, ECM–receptor molecules, and other regulators of RhoA outcompete any activation of YAP signaling that force triggers.

## 4 DISCUSSION

Here, we model flow in the lymphatic vasculature to evaluate the effects of fluid biomechanical force on breast cancer cell behavior and intracellular signaling. WSS altered cell taxis differentially in breast cancer cell lines. Despite correlation between velocity of cell movement and YAP nuclear localization, YAP was dispensable for the motility response to flow and instead suppressed migration. Together with data from our prior report, these findings suggest that mechanotaxis is a response shared by prostate and breast cancer cells but that profound differences in how force sensing is translated are dictated by cell intrinsic determinants that include adhesion interactions and RhoA‐ROCK. A growing body of evidence indicate that mesenchymal movement is characterized by elevated Rac1 activity, growth factor stimulation, integrin engagement, MMP activation, inflammation, and interaction with stiff ECM.^68^ In contrast, amoeboid motility is associated with MMP inhibition, integrin inhibition, and soft ECM. Our data support a role for biomechanical force in transition of cells to an amoeboid mode of motility, dependent upon intrinsic properties of the cell's adhesome and other mechanotransduction machinery.

Breast cancer subtype and genetics likely influence motility response to WSS. In the present study, we examined taxis in two ER^+^ breast cancer lines, one ER^+^ PR^+^ line, three triple negative lines, and one immortalized line. Among these lines, motility response to flow did not correlate with breast cancer subtype or ER/PR expression. In contrast, a prior report showed that ERα^−^ cells exhibit increased amoeboid invasive features in association with downregulation of vinculin transcription and reduced cell–cell and cell–ECM adhesion.^78^ Despite lack of a connection to cancer subtype in our data, this report does support that weak or transient adhesions to the microenvironmental substrate can facilitate amoeboid migration. Amoeboid migration appeared to dominate as a mode of flow‐induced motility and positive responders shared in common a dramatic downregulation of genes encoding adhesion molecules and various components of the ECM. It is notable that lymphatic‐like force could initiate a transition to an amoeboid cell state, as the physical cues regulating this event are still poorly understood.^77^ Interestingly, HCC1806 and HCC1187 engaged preferentially in intracluster cell–cell interactions. Another report demonstrated that Rho/ROCK signaling increases E‐cadherin containing cell–cell adhesions and promotes directional movement of cell sheets,^79^ suggesting that different modes of motility, including collective cell migration, could be engaged by flow. We cannot exclude the possibility that the current experimental platform lacked necessary biomimetic extracellular cues to accurately model egress routes from primary tumors; nevertheless, in a similar platform, prostate cancer cells appear to employ distinct force sensing mechanisms. In future studies, it will be important to examine response of other cell lines with different metastatic properties to evaluate sensitivity and response to WSS, as well as integrate more complex cellular and matrix components that better recapitulate the tumor microenvironment.

A more comprehensive analysis should also include examination of the mechanosensors that mediate motility via RhoA‐ROCK‐myosin II. It could be that the level of expression, sensitivity, or activity of these sensors differ or are modified to tune response or desensitize cells to force. Cell adhesion molecules responsible for homophilic and heterophilic cell–cell interactions such as E‐cadherin are implicated in suppression of breast cancer metastasis to the bone.^80^ E‐cadherin is also known to sequester YAP in the cytoplasm, thus is a critical modifier of YAP‐TEAD transcriptional activity and invasive phenotype.^64^ In addition, other likely sensors include integrins, as their activation state has been linked to cytoskeletal tension^81^ and integrins specific to various types of ECM have established roles in motility response of breast cancer cells to chemical cues.^82^ Integrins regulate the focal adhesion assembly required for actin filament development and elongation. Coordinated adhesion and movement of the cell's leading and trailing edges require contraction of actin filaments, which contribute to migration by myosin II, predominantly through Rho and ROCK.^60^ In the context of physical force caused by confinement, contractility of the actomyosin cytoskeleton via RhoA can be integrin dependent or independent. Confinement can stimulate pronounced rearward cortical flows that result in mesenchymal to amoeboid transition in a variety of cell types, including cancer, in adhesive and nonadhesive 2D and 3D environments.^58^, ^59^ Another pathway that can act independently of integrins, which modifies actomyosin contractility is calcium signaling, another top upregulated KEGG pathway identified in our study. In the presence of compressive force, nuclear deformation contributes to an influx of calcium into the cytosol which triggers cPLA2‐dependent arachidonic acid production and recruitment of myosin II to the actin cortex.^83^, ^84^ Thus, although our data suggest profound differences in cell–cell and cell–ECM adhesion molecules between those cells that adopt a highly motile amoeboid phenotype, we cannot exclude roles for other signaling pathways that similarly dictate cytoskeletal dynamics.

Our data show that WSS alters velocity of cell movement but provides no directional information in our cell culture model; cells uniformly disperse in a radial pattern unlike migration toward a chemokine. The pattern of dispersal is distinct from early reports of migration of endothelial cells in the direction of flow, which was mediated by focal adhesion kinase activation at the leading edge.^85^ These data are, however, consistent with observations of random motility of glioma cells in response to increasing ECM rigidity.^86^ Other chemical and mechanical features of the microenvironment are likely required to enable directional sensing and polarity of movement.^87^, ^88^, ^89^ Immune cell trafficking in the lymphatics is influenced by spatial cues provided by CCL21 secretion and reorganization of PECAM1 and VE‐Cadherin on lymphatic endothelial cells.^90^, ^91^ Similarly, cancer cells rely on gradients of dissolved and surface‐attached chemicals, termed chemotaxis and haptotaxis, respectively. Insulin‐like growth factor I and the chemokine CXCL12 and its receptor, CXCR4, are important for directional migration of breast cancer cells.^82^, ^92^ Both CXCL12 and CXCR4, along with numerous other cytokines and their receptors, were strongly upregulated in positive responders. Recently reported was evidence that amoeboid cancer cells secrete a complex set of factors to support invasion, immune evasion, and endothelial permeability. Additionally, amoeboid migration is perpetuated by cytokine signaling mediated by STAT3 and NF‐κB.^77^ ECM presents growth factors, peptide mediators, and heterogeneous physical features comprised of fibronectin clusters, fibrillar meshworks of collagen, and other filamentous or cable‐like geometries which could serve to guide cancer cells through egress routes from the primary tumor.^93^ Matrix or cell rigidity could also influence polarity of movement, as cells have been shown to preferentially migrate toward rigid environments in a process called durotaxis.^94^ Cell rigidity has also been shown to modulate rolling velocities under physiologic flow conditions.^95^ Although we included the most abundant matrix protein in the ECM—collagen—in our microfluidic device, the structure and other physico‐chemico‐biological properties of collagen are complex and its remodeling is critical for metastatic dissemination of tumor cells.^96^ More sophisticated models will be needed to fully understand the interactions between mechanical and biochemical cues within and surrounding the tumor that dictate tumor and stromal cell behavior.^9^

Collectively, our studies suggest that lymphatic flow provides cancer cells with biomechanical cues that could be leveraged to navigate away from primary tumor sites and spread throughout the body. The force generated by fluid flow regulates cellular behaviors that are fundamental to the process of metastasis, including taxis. Cell movement is required for tumor cell dissemination into healthy tissue, but also critical are other determinants that drive metastatic potential, such as phenotypic plasticity, immune evasion mechanisms, autophagy, anoikis resistance, and metabolic reprogramming. Thus, future studies should seek a comprehensive examination of behavioral and molecular responses to mechanosensing in multiple lineages and subtypes of cancers, with attention to deliberate selection of cell lines to best model the contrast between poorly metastatic and highly metastatic cancers.

## CONFLICT OF INTEREST

The authors have declared explicitly that there are no conflicts of interest in connection with this article.

## AUTHOR CONTRIBUTION

A.M. and M.F.D. performed experiments, analyzed data, and wrote the manuscript. M.L. analyzed the data and edited the manuscript. A.E. edited the manuscript. A.Z., P.D.H., and L.T.O performed experiments. J.P.H. and J.M.L. guided study design and supplied critical reagents. H.J.L. and P.L.W. conceived of the study, analyzed the data, wrote the manuscript, and directed the research.

## Supporting information

Fig S1‐S6

Supplementary Material

Supplementary Material

Supplementary Material

Supplementary Material

Supplementary Material

Supplementary Material
