# Supplementary material for: RhoA‐ROCK competes with YAP to regulate amoeboid breast cancer cell migration in response to lymphatic‐like flow
Source: FASEB Bioadv. 2022 Feb 14;4(5):342–61. doi: 10.1096/fba.2021-00055 (PMC9065582; doi:10.1096/fba.2021-00055)
Supplement: Supplementary file 1 — Fig S1‐S6 [file FBA2-4-342-s005.pdf]

Figure S1

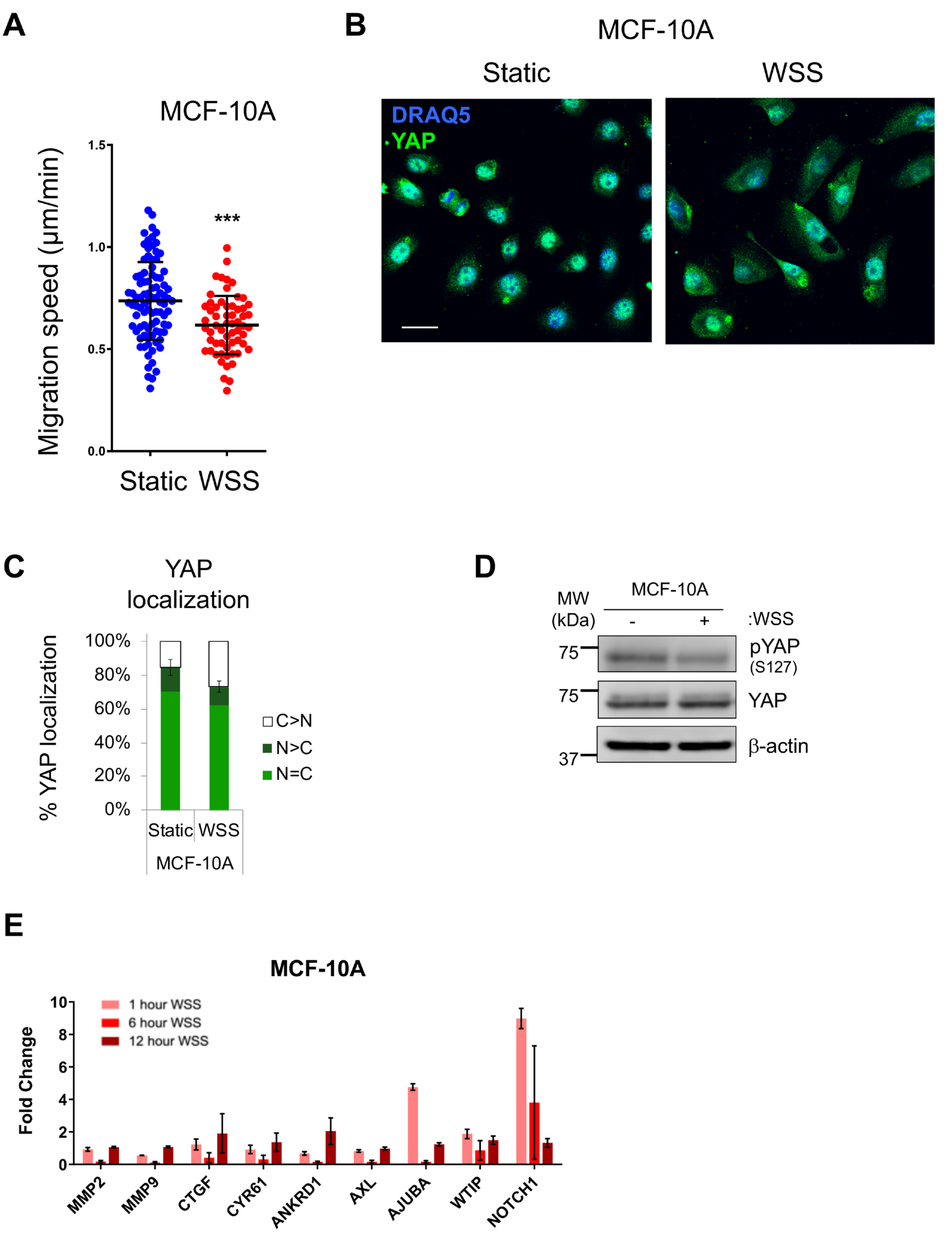

Figure S1. MCF-10A non-tumorigenic immortalized breast cell line responds negatively to flow

(A) Migration speed of MCF-10A cells decreases under WSS. (B, C) Flow induces localization of YAP to the cytoplasm. Scale bar represents 25 μm. (D) YAP immunoblotting shows that flow reduces pYAP levels in

MCF-10A. (E) Transcript levels of genes used as readouts of YAP and/or TAZ activity were measured at 1, 6, or 12 hours after initiation of WSS. Metalloprotease genes *MMP2* and *MMP9* were evaluated as indicators of invasive potential. Error bars represent SEM.

Figure S2

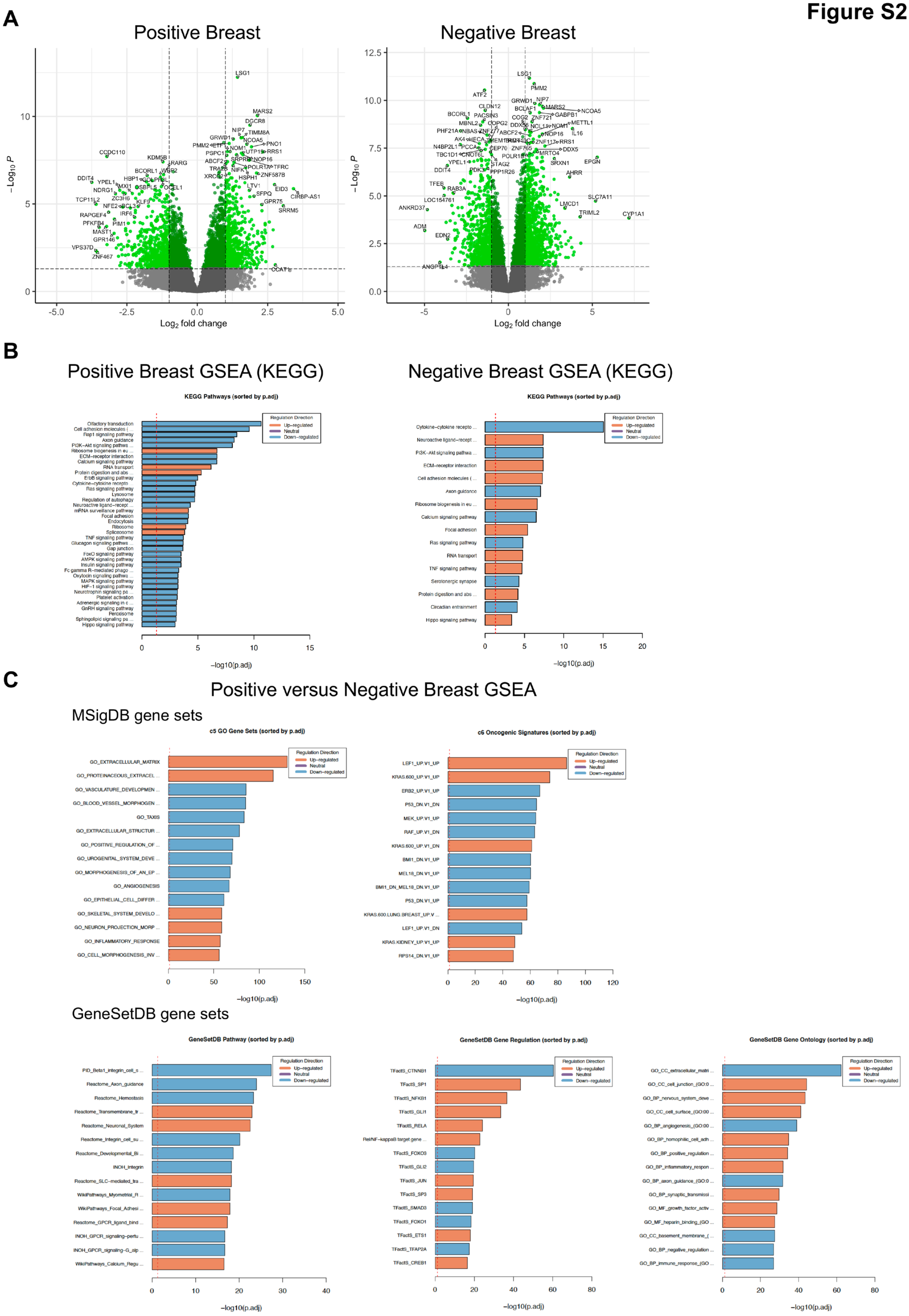
A
Positive Breast
Negative Breast
−Log10 P
Log2 fold change
B
Positive Breast GSEA (KEGG)
Negative Breast GSEA (KEGG)
KEGG Pathways (sorted by p.adj)
Regulation Direction
Up−regulated
Neutral
Down−regulated
−log10(p.adj)
C
Positive versus Negative Breast GSEA
MSigDB gene sets
c5 GO Gene Sets (sorted by p.adj)
c6 Oncogenic Signatures (sorted by p.adj)
GeneSetDB gene sets
GeneSetDB Pathway (sorted by p.adj)
GeneSetDB Gene Regulation (sorted by p.adj)
GeneSetDB Gene Ontology (sorted by p.adj)

Figure S2. Transcriptome and GSEA analysis of flow-exposed versus static cultured breast cancer cells
(A) Differentially expressed genes are shown for single subsets (positive or negative responders) of breast cancer in volcano plots. Cutoffs are indicated by dashed lines and shades of green at p-value<0.05 and $-1<\log2$ fold change<1. (B) Enriched pathways identified from the KEGG database are displayed as bar plots for each breast cancer subset. Contrast reflects change relative to static conditions such that orange bars indicate upregulation with WSS. (C) GSEA pathways significantly enriched from MSigDB and GeneSetDB databases reveal strong signatures of extracellular matrix, integrins, cell junctions, Wnt/LEF, β-catenin regulation, and NF-κB in the positive versus negative breast cancer comparison. Orange bars indicate pathways predicted to be upregulated in positive responders exposed to flow relative to negative responders exposed to flow. Blue bars display pathways predicted to be downregulated.

Figure S3

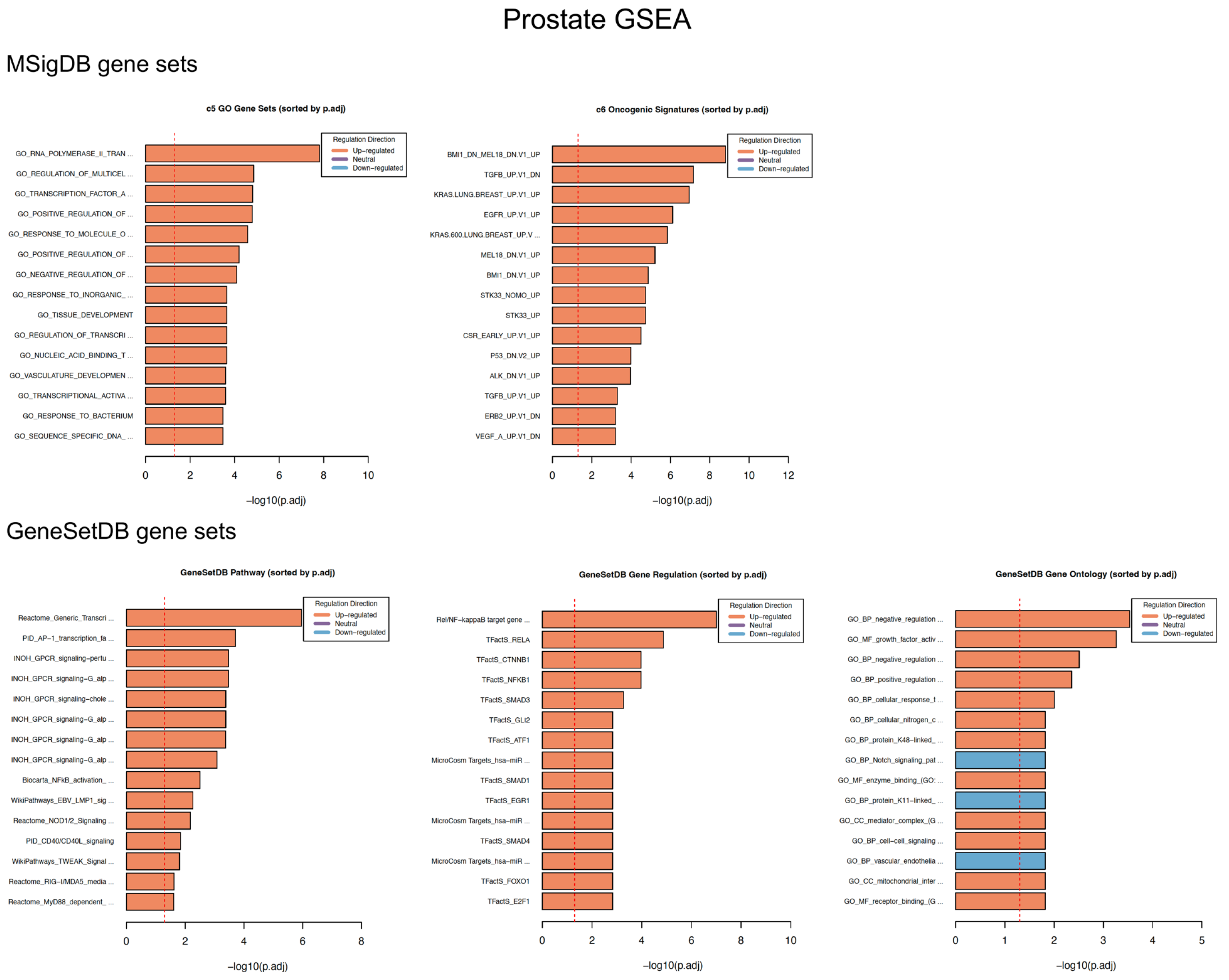

Figure S3. GSEA pathway analysis of flow-exposed versus static cultured prostate cancer cells

Pathways identified from MSigDB and GeneSetDB databases in PC3 prostate cancer cells after WSS exposure suggest enrichment of genes involved in transcription, NF-κB, β-catenin, BMI-1, TGF-β, EGFR, and G-protein coupled receptor signaling.

Figure S4

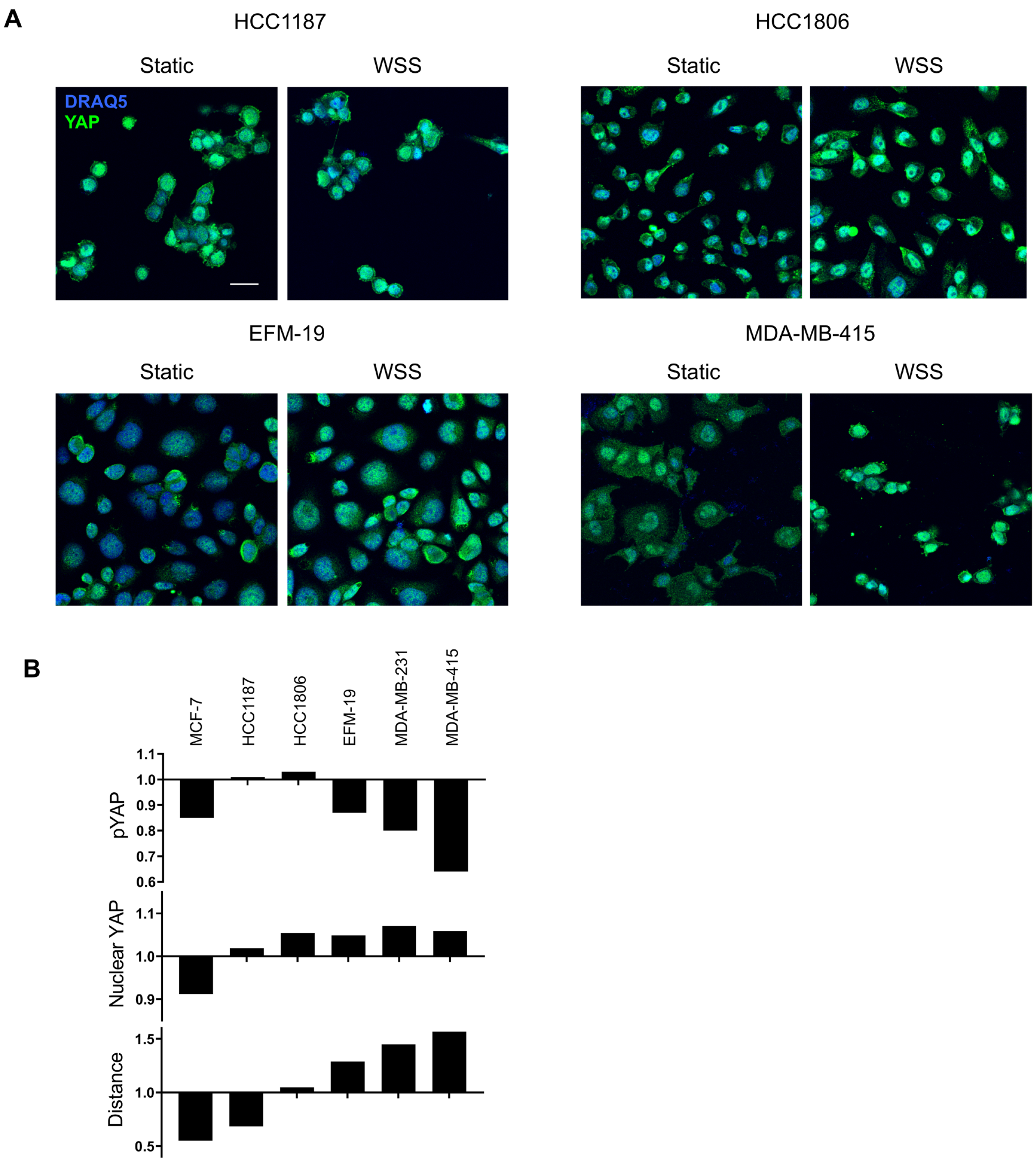

Figure S4. YAP subcellular localization under flow varies across breast cancer cell lines

(A) Photomicrographs of cell lines show differing effects of flow on YAP localization within the nucleus and cytoplasm. Scale bar represents 25 µm. (B) Fold change in pYAP and nuclear YAP after WSS are plotted relative to static. Nuclear YAP localization in WSS conditions is plotted relative to static cultures. Total

distance traveled by cells after WSS exposure is depicted as fold change relative to static migration. Breast lines with increased cell motility under flow had increased YAP nuclear localization.

Figure S5

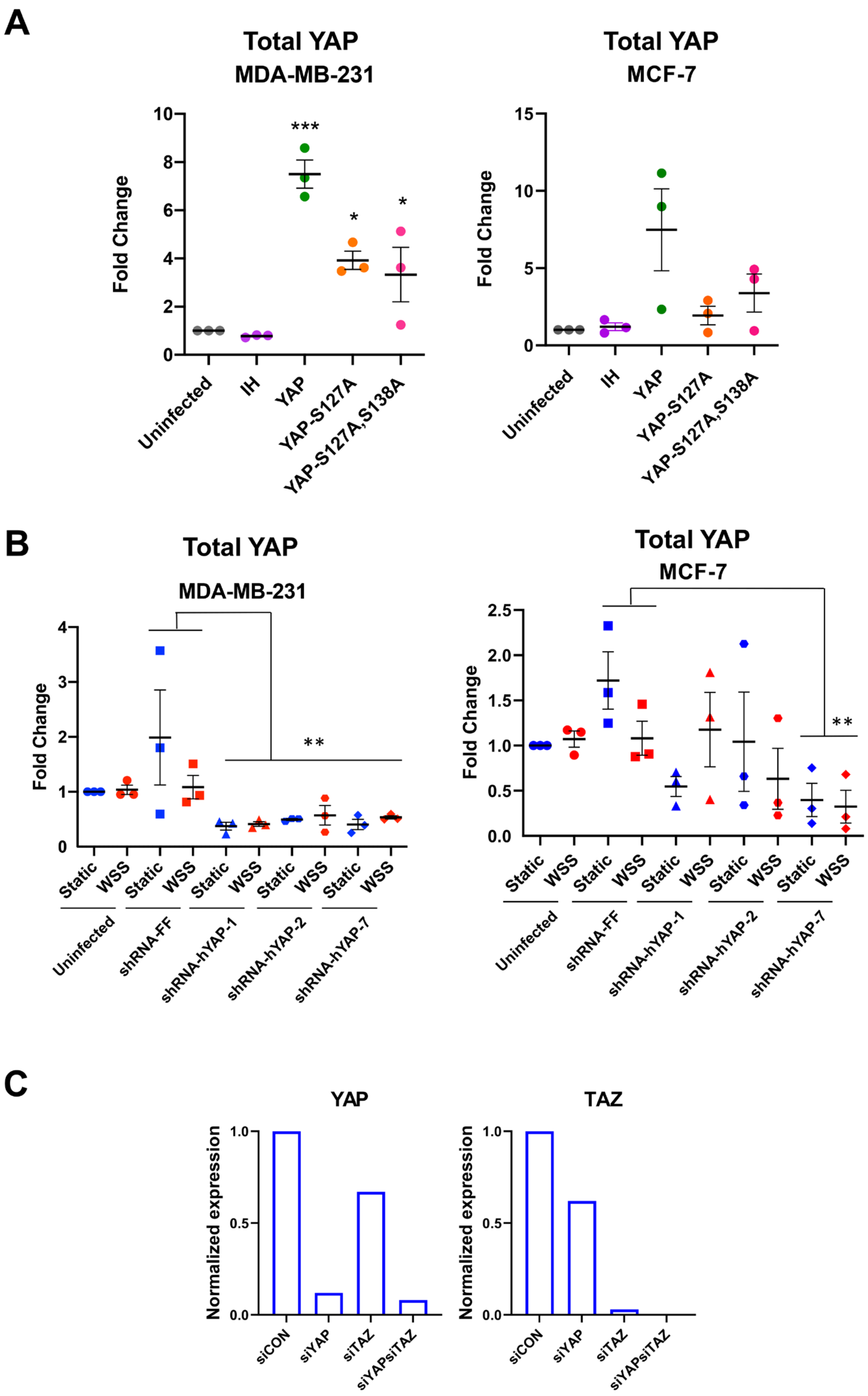

Figure S5. Overexpression and knockdown efficiency of YAP and TAZ

(A) Ectopic expression of YAP by retrovirus was confirmed to result in elevated total YAP protein levels in MDA-MB-231 and MCF-7 cells (One-way ANOVA, *p<0.05 and ***p<0.001 relative to uninfected control). Expression of YAP after (B) shRNA knockdown or (C) YAP and TAZ after siRNA knockdown was confirmed

by immunoblot (One-way ANOVA, **$p<0.01$ relative to uninfected control). Error bars on immunoblot quantification indicate SEM.

Figure S6

A

Cell Adhesion Molecules (CAMs)

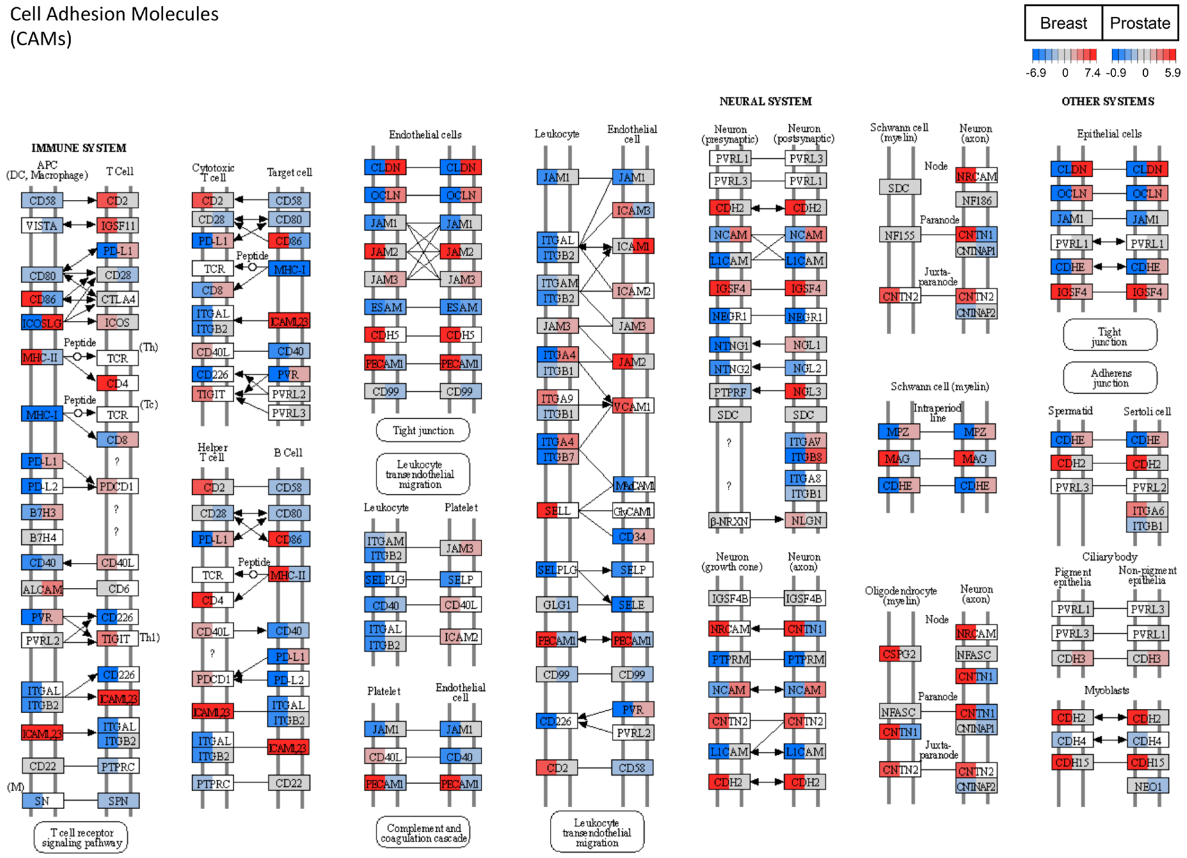

B

ECM-Receptor Interaction

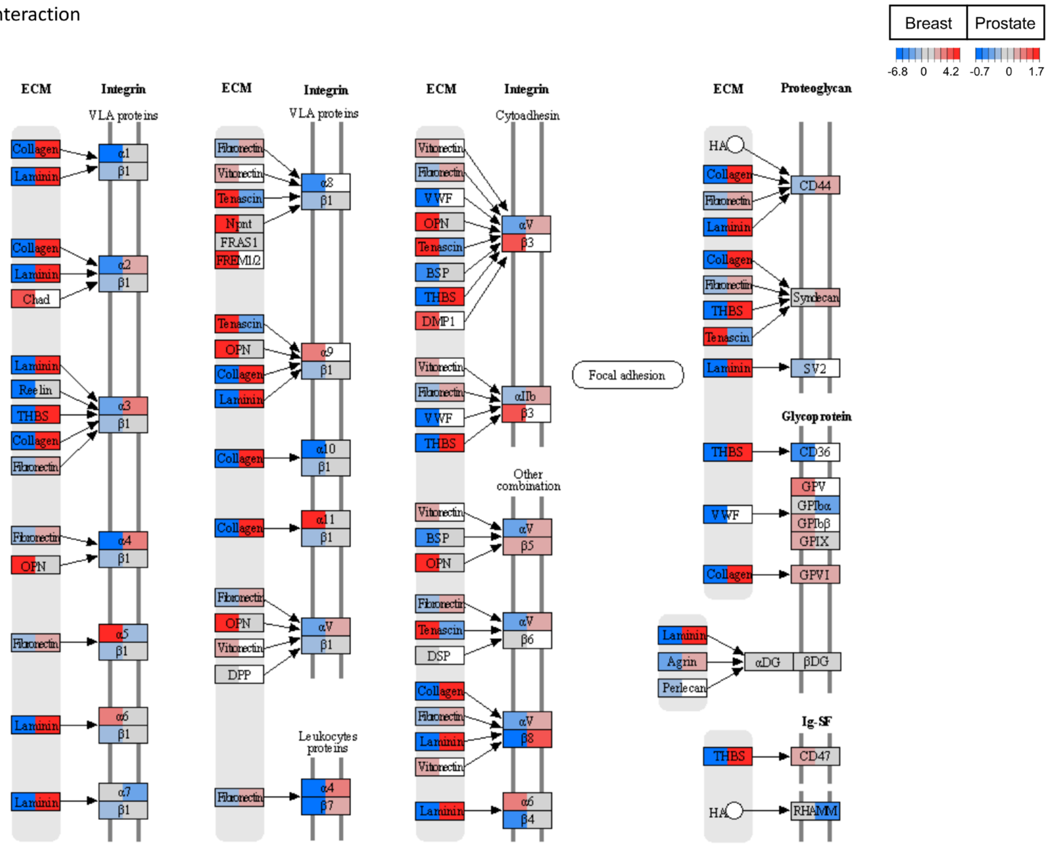

Figure S6. Cell adhesion molecules and ECM-receptor interaction pathways are regulated differently based upon response to flow.

(A) KEGG pathway for cell adhesion molecules indicates fold change in expression of various proteins presented on the cell surface for interactions between cells. (B) Matrix proteins and cell surface receptors that mediate ECM-receptor interactions are displayed to show log fold change in gene expression induced by flow in positive-versus-negative breast cancer subsets and in prostate cancer cells. Color in left half of node corresponds with breast and color in right half with prostate.
